# Spatial Immune Profiling and AI-Based Classifiers Identify Predictors of BCG Therapy Outcomes in High-Risk Non-Muscle-Invasive Bladder Cancer

**DOI:** 10.3390/cancers18060938

**Published:** 2026-03-13

**Authors:** Melinda Lillesand, Marie Austdal, Jakub Mroz, Ivar Skaland, Einar Gudlaugsson, Florus C. de Jong, Tahlita C. M. Zuiverloon, Kjersti Engan, Emiel A. M. Janssen

**Affiliations:** 1 Department of Pathology, Stavanger University Hospital, 4011 Stavanger, Norway; ivar.skaland@sus.no (I.S.); einar.gudbjorn.gudlaugsson@sus.no (E.G.); emilius.adrianus.maria.janssen@sus.no (E.A.M.J.); 2 Department of Chemistry, Bioscience and Environmental Engineering, University of Stavanger, 4021 Stavanger, Norway; 3 Section for Biostatistics, Department of Research, Stavanger University Hospital, 4011 Stavanger, Norway; marie.austdal@sus.no; 4 Department of Electrical Engineering and Computer Science, University of Stavanger, 4021 Stavanger, Norwaykjersti.engan@uis.no (K.E.); 5 Department of Urology, Erasmus MC Cancer Institute, Erasmus University Medical Center, 3015 GD Rotterdam, The Netherlands; f.c.dejong@erasmusmc.nl (F.C.d.J.); t.zuiverloon@erasmusmc.nl (T.C.M.Z.)

**Keywords:** high-risk non-muscle-invasive bladder cancer, Bacillus Calmette–Guérin, imaging mass cytometry, spatial proteomics, tumor microenvironment, treatment response, progression, biomarkers, risk stratification, BCG response subtypes

## Abstract

**Background/Objectives**: High recurrence rates and intensive lifelong surveillance make bladder cancer among the costliest malignancies to treat. Although Bacillus Calmette–Guérin (BCG) immunotherapy is the standard treatment for high-risk non-muscle-invasive bladder cancer (NMIBC), up to 50% of patients fail to respond, and predictive biomarkers are lacking. Molecular profiling has established three BCG response subtypes (BRS1–3), with BRS3 characterized by an immunosuppressive, BCG-resistant phenotype; however, these features have not been validated at single-cell spatial resolution. **Methods**: We applied imaging mass cytometry (IMC) to 82 BCG-treated high-risk NMIBC samples and performed (i) single-cell IMC with unsupervised clustering to identify phenotypic cell clusters and quantify cluster abundances and (ii) a convolutional neural network-based gated attention multiple instance learning model trained on IMC images (IMC-GA-MIL) to predict BCG response. Cluster abundances were summarized using II (immune composition within the immune compartment), TT (tumor phenotypic composition), and IT (immune/stromal abundance relative to tumor cells) indices. **Results**: Single-cell IMC identified 18 distinct phenotypic cell clusters. In BCG responders, immune cells localized within the tumor compartment were enriched and independently protective (HR 0.67, 95% CI 0.49–0.92). BCG nonresponse was associated with a higher abundance of fibroblast-dominant clusters relative to tumor cells (IT index). Plasma cell-dominant clusters were the strongest predictors of progression (II index HR 2.28, 95% CI 1.37–3.79; IT index HR 1.25, 95% CI 1.06–1.48). The IMC-GA-MIL model predicted BCG response with 90% accuracy (9/10) and identified myeloid- and T-cell-associated marker patterns involving CD14, CD11b, CD68, CD8, and FOXP3 as the most informative contributors. **Conclusions**: Spatial single-cell profiling and IMC-GA-MIL identify spatial immune and stromal features associated with BCG failure. However, findings from both analyses should be considered exploratory and will require validation in larger, independent cohorts.

## Simple Summary

High-risk non-muscle-invasive bladder cancer (NMIBC) frequently recurs and may progress, requiring intensive surveillance. Bacillus Calmette–Guérin (BCG) immunotherapy is the standard treatment for high-risk disease; however, reliable biomarkers of treatment failure are lacking. We analyzed 82 high-risk NMIBC tumors treated with BCG using Hyperion imaging mass cytometry (IMC) and evaluated the data using two complementary approaches: spatial single-cell phenotyping and an IMC-specific AI-based gated attention multiple instance learning model (IMC-GA-MIL) to predict BCG response. Using single-cell IMC data, we characterized tumor, immune, and stromal phenotypes and examined their associations with BCG response and survival. BCG nonresponse was associated with a tumor microenvironment enriched in fibroblasts and plasma cells, whereas BCG response was associated with immune cells localized within tumor regions. The IMC-GA-MIL model identified marker patterns consistent with immunosuppressive biology as key predictive features of BCG response.

## 1. Introduction

Bladder cancer is one of the most expensive cancers to treat because of high recurrence rates and the need for intensive long-term surveillance [1]. Most cases (approximately 75%) are non-muscle-invasive bladder cancer (NMIBC), of which approximately 30% progress to muscle-invasive bladder cancer (MIBC) [2,3]. The European Association of Urology (EAU) stratifies NMIBC into low-, intermediate-, high-, and very high-risk groups based on tumor stage, grade, size, number, presence of carcinoma in situ (CIS), recurrence history, lymphovascular invasion (LVI), and histological variants [4,5]. Since 1976, Bacillus Calmette–Guérin (BCG) immunotherapy has been the standard adjuvant treatment for intermediate- and high-risk NMIBC following transurethral resection of bladder tumor [4,6]. BCG is typically administered as a six-week induction course followed by maintenance instillations for up to three years [2,4]. However, up to 50% of patients fail to respond. Current EAU risk stratification has limited predictive power because of inconsistent staging and grading and an inability to capture tumor heterogeneity [7,8,9,10]. Therefore, early identification of BCG nonresponders is critical for timely initiation of alternative treatments, such as radical cystectomy or targeted therapies [8,11].

To improve risk stratification, de Jong et al. performed bulk transcriptomic profiling of high-risk NMIBC and identified three BCG response subtypes: BRS1, BRS2, and BRS3 [8]. BRS3 tumors exhibit an immunosuppressive profile and are associated with shorter recurrence-free survival (RFS) and progression-free survival (PFS) [8]. In matched pre- and post-BCG samples from nonresponders, tumors often shift from BRS1 or BRS2 to BRS3 [8]. However, the cellular composition underlying these transcriptomic subtypes has not been fully characterized at single-cell resolution.

BCG therapy recruits both innate and adaptive immune cells to the bladder tumor microenvironment (TME) [12]. Nevertheless, immunosuppressive cell phenotypes within the TME are consistently associated with poor treatment outcomes. Suppressive innate immune populations, including N2-type neutrophils and M2-type macrophages (CD163^+^ CD204^+^), have been implicated in tumor growth and angiogenesis [13,14,15,16,17]. Myeloid-derived suppressor cells (MDSCs), comprising granulocytic (CD11b^+^ CD15^+^ HLA-DR^−^ CD14^−^), monocytic (CD11b^+^ CD14^+^ HLA-DR^−^ CD15^−^), and early-stage (CD11b^+^ HLA-DR^−^) subsets, are immunosuppressive and have been linked to BCG failure [18,19,20]. Adaptive immunity is critical for therapeutic efficacy; effective BCG responses require a Th1-dominant environment, including cytotoxic CD8^+^ T cells and natural killer (NK) cells [21,22,23]. By contrast, Th2-skewed TMEs enriched in FOXP3^+^ regulatory T cells (Tregs), Th2 CD4^+^ T cells, and B cells are linked to BCG failure [24,25,26]. High CD38 expression has also been associated with immune dysfunction and poor treatment outcomes in bladder cancer [27]. Moreover, high expression of immune checkpoints, such as PD-1 and VISTA, together with exhausted CD8^+^ T cells, may further limit anti-tumor activity [28,29]. Fibroblasts, which normally provide tissue support and repair, can acquire a cancer-associated fibroblast (CAF) phenotype in tumors. Vimentin^+^ CAFs secrete immunoregulatory factors and are linked to reduced BCG response in NMIBC [30].

Bladder tumors are highly heterogeneous and contain diverse cell types that co-express multiple proteins in structured microenvironments, warranting multi-marker characterization of cellular phenotypes [31]. Conventional immunohistochemistry (IHC) detects only a few markers per tissue section, whereas imaging mass cytometry (IMC) measures up to 40 markers simultaneously at single-cell resolution, preserving spatial context. Computational pathology analyzes digital pathology images using methods from segmentation-based quantification to deep learning-based prediction [32]. IMC data are commonly analyzed using single-cell analysis, a segmentation-based workflow that identifies individual cells and quantifies marker expression and spatial relationships. In MIBC, Feng et al. used single-cell IMC to characterize the TME and identified 21 cellular clusters, including a stem-like tumor cell population associated with advanced stage and poor survival [33]. In a subsequent study, the same group analyzed 40 MIBC tumors across 185 regions of interest and described distinct fibroblast subtypes. One subtype, defined by α-SMA^+^, collagen I^+^, vimentin^+^, and CD90^+^ expression, was enriched at the tumor leading edge and associated with abnormal vasculature and poor prognosis [34]. Recent spatial single-cell studies have also adopted relative, compartment-aware measures to assess immune infiltration rather than absolute counts, enabling consistent comparison across samples by correcting for variation in tumor size and cellularity [31,35]. To our knowledge, image-based deep learning prediction of BCG treatment response in bladder cancer remains limited. Using routine hematoxylin and eosin (H&E) whole-slide images, Fuster et al. applied convolutional neural networks (CNNs) with attention-based multiple instance learning (MIL) to predict recurrence and BCG treatment response in NMIBC, achieving modest prognostic performance (area under the receiver operating characteristic curve [ROC-AUC] ≈ 0.68 for BCG response) [36].

In this study, we aimed to identify immune and stromal features within the TME associated with BCG treatment response using IMC in tumors from the high-risk NMIBC cohort reported by de Jong et al. [8]. We applied two complementary analytical strategies: (i) single-cell IMC analysis to characterize TME cell composition and spatial organization and to assess associations with BCG response and BRS subtypes; and (ii) an IMC-specific CNN-based gated attention multiple instance learning (IMC-GA-MIL) model to predict patient-level BCG treatment response.

## 2. Materials and Methods

### 2.1. Patient Data

A total of 427 formalin-fixed, paraffin-embedded (FFPE) tissue microarray (TMA) samples from patients diagnosed with high-risk NMIBC between 2000 and 2018 were identified from four Dutch hospitals and Stavanger University Hospital (Norway). Given the cost and time demands of IMC, a representative subset was selected based on histopathological review of H&E-stained sections to ensure adequate tissue quality and the presence of detrusor muscle, which is required for accurate staging under EAU guidelines. This process yielded 88 samples (40 responders and 48 nonresponders) for single-cell IMC analysis. Following IMC acquisition, six samples were excluded because of poor nuclear staining or consistently low marker quality across multiple channels, resulting in 82 samples (37 BCG responders and 45 nonresponders) for analyses of BCG treatment response. Clinical follow-up data for RFS, PFS, and BRS subtype classification were available for 79 patients, who were included in survival and BRS subtype association analyses.

For prediction of BCG response using the IMC-GA-MIL model, a subset of 69 patients with high-quality IMC images was selected to minimize technical noise and facilitate robust feature extraction and stable model training. This subset maintained a balanced distribution of BCG responders (*n* = 33) and nonresponders (*n* = 36). Patients were randomly assigned to a training set (*n* = 59; 28 BCG responders and 31 nonresponders) and an independent test set (*n* = 10; 5 BCG responders and 5 nonresponders). Model optimization was performed on the training set, and performance was evaluated on the test set (Table 1). To ensure independence between training and test data, the train–test split was performed strictly at the patient level, and no image patches or derived features from test-set patients were used during model development.

Exclusions at each stage were small in number, were based solely on IMC technical quality criteria (e.g., tissue integrity, staining performance), were not informed by clinical characteristics or BCG response status, and resulted in responder/non-responder proportions that remained comparable before and after exclusion.

### 2.2. Clinical Characteristics and Endpoints

Histopathological and clinical features were retrospectively recorded for all cases from routine pathology reports and clinical records, including sex, smoking status, tumor focality, CIS, LVI, histological variant, T1 substaging, and EAU risk group [8]. BCG response was defined as the absence of HG recurrence after at least 5 of 6 induction instillations and at least 9 maintenance instillations according to the SWOG BCG protocol. BCG failure was defined as muscle-invasive progression, persistent T1 HG disease after induction, or HG recurrence following adequate BCG therapy [4,8].

Clinical follow-up was conducted in accordance with EAU guidelines, and tumors were classified as high-risk or very high-risk based on EAU criteria [4,8]. RFS was defined as the interval from high-risk NMIBC diagnosis to the first biopsy-proven recurrence. PFS was defined as the interval from high-risk NMIBC diagnosis to the development of MIBC or metastatic spread (lymph node or distant) [8].

### 2.3. IMC Data Analysis Pipelines

**Figure 1:**
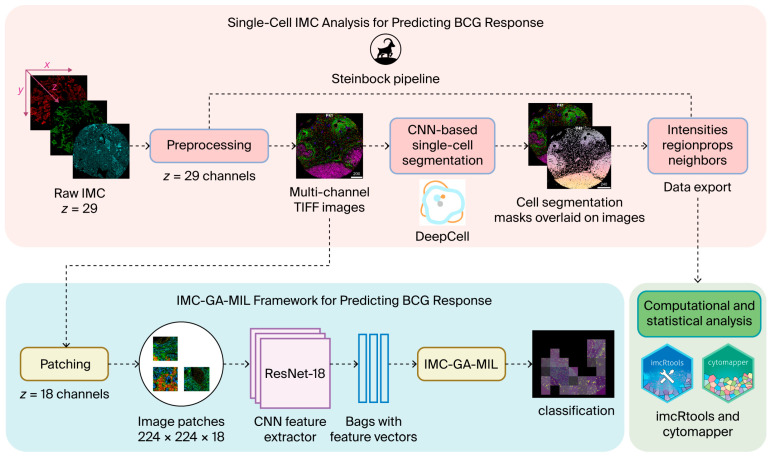
Overview of single-cell IMC analysis and IMC-GA-MIL model for predicting BCG response. IMC data consist of spatially resolved, multiplexed images in which the x–y plane represents tissue spatial coordinates at 1 µm × 1 µm resolution, and the z dimension represents marker channels. In this study, raw IMC images comprised 29 markers and were analyzed using two pipelines. The single-cell IMC analysis (**upper panel**) used all 29 markers for cell segmentation and extraction of per-cell quantitative features using the Steinbock pipeline. In parallel (**lower panel**), an IMC-GA-MIL model was applied using a subset of 18 selected markers. Images were divided into fixed-size patches (224 × 224 pixels), and feature vectors from all patches belonging to the same patient were grouped into a single bag. Patch-level feature vectors were weighted by an attention mechanism and aggregated to generate a patient-level prediction.

IMC data were used as input for two analytical pipelines: (i) a single-cell IMC analysis based on cell segmentation and quantitative marker expression and (ii) an IMC-specific gated attention and CNN-based learning framework (Figure 1).

### 2.4. IMC Antibody Panel and Tissue Staining

IMC was performed on FFPE TMA sections from NMIBC specimens using a validated antibody panel. Pre-conjugated metal-tagged antibodies were obtained from Standard BioTools™ (South San Francisco, CA, USA). Additional antibodies were conjugated in-house from BSA- and azide-free sources (Cell Signaling Technology, Danvers, MA, USA; Abcam, Cambridge, UK) following the manufacturers’ protocols. In-house–conjugated antibodies were validated by conventional IHC, and optimal working concentrations were determined using IMC on control tissue sections.

FFPE TMA sections were deparaffinized, rehydrated, and subjected to antigen retrieval in EDTA buffer (pH 9.0) (Agilent Technologies, Santa Clara, CA, USA) at 96 °C for 30 min. Sections were blocked with 3% bovine serum albumin (Sigma-Aldrich, St. Louis, MO, USA) and incubated overnight at 4 °C with the metal-tagged antibody cocktail. Nuclei were counterstained using a DNA intercalator (Standard BioTools, South San Francisco, CA, USA) prior to IMC acquisition. The initial IMC panel comprised 35 protein markers and two nuclear stains. Following quality control, six markers (CD115, CD141, CD45RO, PD-L1, PD-L2, and LAG-3) were excluded owing to absent or suboptimal signal quality. The final panel used for downstream analyses consisted of 29 protein markers and two nuclear stains (Table 2).

### 2.5. IMC Data Acquisition and Data Format

IMC generates digital multiplexed images in which each pixel encodes signal intensities for multiple protein markers. Each region of interest represented a single patient sample corresponding to one TMA core (~1 mm in diameter). IMC images were represented as 3D arrays with dimensions height × width × channels, where height and width corresponded to the number of pixels covering the TMA core, with each pixel representing a 1 µm × 1 µm tissue area. The channels corresponded to individual protein markers, including 29 protein channels and two nuclear channels.

Raw IMC data were acquired and stored in MCD format, preserving spatial coordinates. These were subsequently converted to multi-channel TIFF images (~1000 × 1000 pixels, approximately 100 MB per TMA core) for downstream single-cell IMC and IMC-GA-MIL analyses. Data quality, including TMA integrity and channel signal intensity, was initially assessed using MCD Viewer (Standard BioTools™, South San Francisco, CA, USA).

### 2.6. Single-Cell IMC Analysis

**Figure 2:**
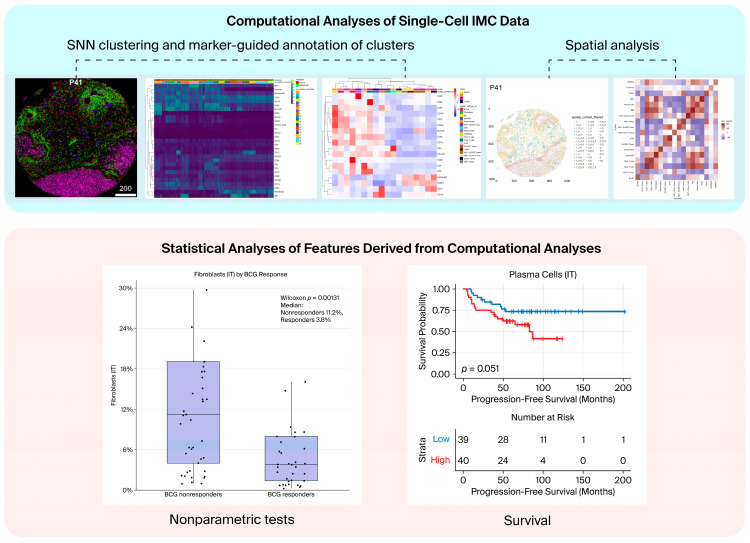
Overview of the single-cell IMC analysis workflow. Single-cell IMC data were analyzed using a two-step approach. (**Upper panel**): Computational analyses of single-cell IMC data, including shared nearest neighbor graph-based clustering followed by manual annotation of phenotypic cell clusters based on marker expression, as well as spatial analyses to assess cell localization and cell–cell interactions within the tumor microenvironment. (**Lower panel**): Statistical analyses of features derived from the computational analyses, including nonparametric group comparisons and survival analyses to evaluate associations with BCG treatment response, BCG response subtypes (BRS), and patient outcomes.

Single-cell IMC analysis was performed to quantify cellular phenotypes and spatial relationships within the TME and to evaluate their associations with BCG response and BRS subtypes (Figure 2).

#### 2.6.1. Preprocessing and Single-Cell Segmentation

IMC images were processed using the Steinbock IMC workflow (v0.16.3), as previously described [37]. Briefly, raw MCD files were converted to multi-channel TIFF images, and hot pixels were removed. Single-cell segmentation was performed using the Mesmer algorithm (DeepCell), generating single-cell masks in which each cell was assigned a unique integer label [37,38]. From these segmentation masks, single-cell features were extracted, including mean marker intensities and morphological properties (cell area, centroid coordinates, major and minor axis lengths, and eccentricity). Spatial neighborhood relationships were computed from centroid-to-centroid distances to construct k-nearest-neighbor (kNN) graphs, which were stored as directed edge lists. All processing steps were executed in a steinbock Docker container (v0.16.3) to ensure reproducibility and fixed software versions [37,38]. Segmentation quality was visually assessed using napari-imc (Swiss Institute of Bioinformatics, Lausanne, Switzerland) by overlaying segmentation masks on marker channels to evaluate alignment and segmentation accuracy [37].

#### 2.6.2. Computational Analysis of Single-Cell IMC Data

**Figure 3:**
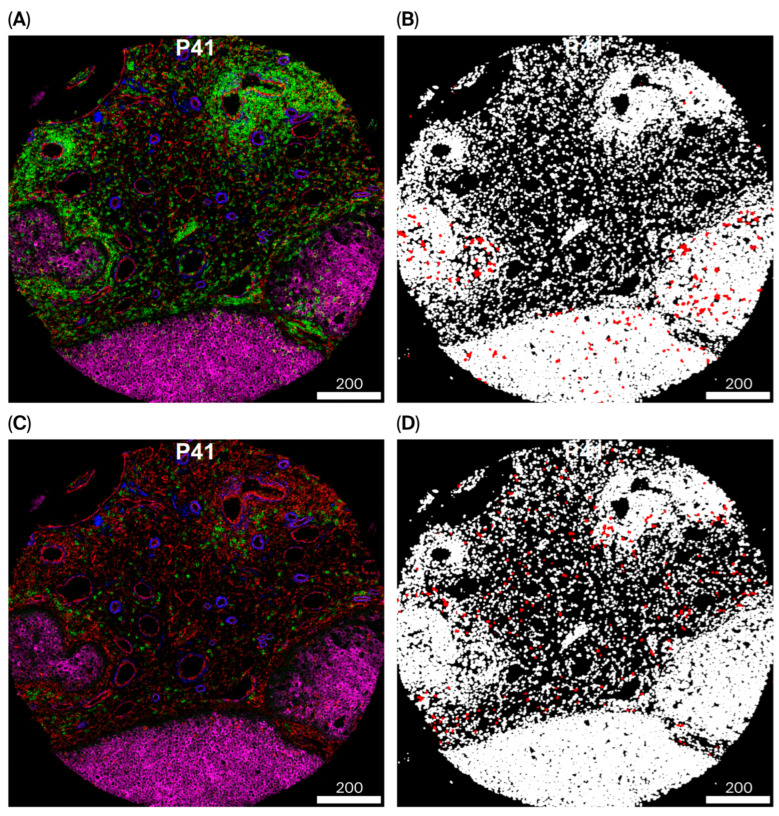
Representative IMC image (P41) of tumor tissue showing marker expression and corresponding cell annotations. (**A**) Pixel-based visualization of vimentin (red), CD45 (green), SMA (blue), and pan-keratin (magenta). (**B**) Segmentation-based cell annotation highlighting immune cells localized within the tumor compartment (red) overlaid on all segmented cells (white). (**C**) Pixel-based visualization of vimentin (red), CD38 (green), SMA (blue), and pan-keratin (magenta). (**D**) Segmentation-based cell annotation highlighting plasma cells (red) overlaid on all segmented cells (white). Scale bar, 200 µm.

Computational analyses, including unsupervised clustering and spatial analyses, were performed on IMC-derived single-cell data in R (v4.5.1) using Bioconductor packages, including imcRtools (v1.14.0). Marker intensities were asinh-transformed, and batch effects across patients were addressed using mutual nearest neighbors correction with batchelor::fastMNN, which generated an integrated low-dimensional embedding for downstream analyses. Shared nearest-neighbor graph-based clustering with Louvain community detection was performed using scran/bluster. Clustering performance was evaluated using average silhouette width and neighborhood purity. Clusters were manually annotated based on marker expression profiles visualized using heatmaps and UMAP. Annotations were further confirmed by inspection of IMC images with cytomapper and by comparison with corresponding H&E-stained sections in consultation with a pathologist [37,39,40] (Figure 3 and Figure S1). Although IMC-derived phenotypes were qualitatively reviewed against adjacent H&E sections, the IMC and H&E slides did not overlap sufficiently to allow precise cell-level correspondence. Therefore, no systematic quantitative histopathological correlation was performed.

Cellular neighborhoods (CNs) were defined using centroid-based kNN graphs (k = 20) by aggregating mean marker expression across local neighborhoods, followed by k-means clustering of the resulting neighborhood profiles (10 clusters). Broader spatial contexts (SCs) were derived analogously using larger kNN graphs (k = 40). k values were selected to capture local cellular neighborhoods and broader spatial contexts, consistent with common practice in kNN-based spatial analyses of multiplex imaging data. Pairwise interactions between cell clusters were assessed using the imcRtools testInteractions function to assess whether clusters co-localized more frequently than expected by chance within each image. The resulting clusters and spatial features were used for statistical analyses assessing associations with BCG response and BRS subtypes [37,39].

### 2.7. Gated Attention and CNN-Based Learning for Prediction of BCG Treatment Response

We developed a two-stage residual neural network (ResNet)-based MIL framework, termed IMC-GA-MIL, to predict BCG treatment response from IMC data. The framework is tailored to IMC images with a large number of channels (Figure 1). The training procedure consisted of two stages: (i) patch-based training of the feature extractor using only the training set, and (ii) patient-level MIL training with the feature extractor frozen, also using only the training set. No test data were used for model selection or tuning; ResNet-18 was selected a priori as a compact backbone suitable for limited IMC datasets.

The trainable components were intentionally kept small relative to the dataset size. During stage-one feature-extractor training, only the final ResNet-18 classifier layers were unfrozen (57,602 trainable parameters). During stage-two MIL training, the feature extractor was frozen and only the gated-attention module and final classifier were trainable (131,970 parameters).

#### 2.7.1. Image Normalization and Patching

As described in the Patient Data section, the dataset comprised 59 patients in the training set and 10 in the test set. Multi-channel TIFF images generated using the Steinbock IMC workflow were used as input for the IMC-GA-MIL model. Of the 29 protein markers, 18 representative markers were selected a priori to capture major tumor, stromal, and immune phenotypes while maintaining a favorable signal-to-noise ratio and limiting model complexity (Table 3). The resulting 18-channel TMA images were normalized using percentile-based intensity scaling. Pixel values were rescaled to a 0–1 range, and values above the 99.9th percentile were clipped to reduce the influence of extreme outliers [41,42]. Normalized images were divided into fixed-sized patches (224 × 224 pixels) with 50% spatial overlap using the Patchify Python (v0.2.3) library. This overlap ensured inclusion of structures near patch boundaries in multiple patches, reducing edge effects and minimizing information loss. Patches with less than 30% tissue signal were excluded, yielding 2635 and 492 training and testing patches, respectively.

Training data were augmented using 90° rotations (fourfold) and Gaussian noise (σ = 0.001, 0.005, 0.01; fourfold), resulting in 42,160 patches. Fifteen percent of the augmented training patches were randomly selected to form a validation set, which was used during training to monitor classification performance. Patches were converted to 224 × 224 × 18 tensors in PyTorch (v2.5.0) and used as input to the ResNet model, with each channel representing marker intensity. For the AI-based experiments, only weak labels were applied, using image-level labels defining two classes: BCG responder and nonresponder.

#### 2.7.2. Two-Stage IMC-GA-MIL Model for BCG Response Prediction

Stage-one: A CNN ResNet-18 model, pre-trained on ImageNet, was adapted to accept 18 input channels by inflating the first convolutional layer [43,44]. All patches inherited the image-level label, and the model was trained using weakly labeled patches. After training, the classification layer was removed, and the network was used as a frozen feature extractor.

Stage-two: A gated attention-based MIL (GA-MIL) approach was applied for image-level response prediction. In this framework, each IMC image constituted a bag, and patches extracted from that image represented the instances within the bag. Patch-level feature embeddings from each image were aggregated as a weighted average using learnable attention weights, according to the GA-MIL method, in which weights sum to one and are determined by a neural network. The aggregated image embedding was passed through a learnable classifier consisting of a single fully connected layer with a sigmoid output to generate a patient-level prediction (BCG responder vs. nonresponder) using a fixed decision threshold [45].

The number of instances per IMC image bag varied; therefore, bags were zero-padded within each batch to ensure a consistent bag size. Images with fewer than 20 patches and one outlier image were excluded. This resulted in 53 patient bags (27 BCG responders and 26 nonresponders) used for training in stage two. The GA-MIL model was trained using the Adam optimizer (learning rate = 0.0005, batch size = 6) for 100 epochs. The feature extractor from stage one was kept frozen during GA-MIL training because of the limited number of image-level samples.

#### 2.7.3. Assessment of IMC-GA-MIL

Model performance was evaluated using standard binary classification metrics, including sensitivity (recall), positive predictive value (precision), F1 score, and the ROC–AUC. To provide additional uncertainty estimation, we also computed precision–recall (PR) AUC, the Brier score, and the confusion matrix at a 0.5 decision threshold for the independent hold-out cohort. Because bootstrap CIs collapse to 1.00–1.00 in this small, perfectly separated test set, we report the exact 95% Clopper–Pearson binomial CI derived from the 25 pairwise positive–negative comparisons (0.86–1.00). In addition, a 3-fold patient-level cross-validation was conducted exclusively on the training set (with the feature extractor frozen) as an internal robustness check; the independent 10-patient hold-out cohort was not used in cross-validation. Nested cross-validation was not performed because of the limited sample size.

Unfortunately, no external IMC cohort with a comparable marker panel was available for validation; therefore, the IMC-GA-MIL results should be considered exploratory. Repeated resampling or stability analyses were not performed due to the limited dataset size.

### 2.8. Statistical Analysis of Single-Cell IMC Data

Statistical testing was performed for single-cell IMC-derived features, whereas the IMC-GA-MIL framework was evaluated using predictive performance metrics only. Statistical analyses of single-cell IMC data aimed to test whether differences in cell clusters and spatial features were associated with BCG response, BRS molecular subtype, and clinical outcomes. All statistical analyses were performed in R (v4.5.1, Vienna, Austria).

Unsupervised graph-based clustering assigned each cell to a cluster, and the number of cells per cluster was calculated for each patient. Clusters were reviewed for marker expression profiles and assigned to immune, tumor, or stromal phenotypes based on dominant expression patterns. One cluster with non-specific marker expression was classified as undefined [39,40]. Because the absolute numbers of tumor, immune, and stromal cells varied widely across patients, raw cluster counts were not directly comparable. Compartment-based analyses, as described in several high-multiplex imaging studies [31,35,46,47,48,49], quantify immune subsets within the immune compartment, tumor subsets within the tumor compartment, and the immune/stroma-tumor balance. Therefore, we calculated three simple indices for each patient. The II index describes immune subset composition relative to all immune cells and was defined as (immune subset + 0.5)/(all immune + 1). The TT index describes tumor subset composition relative to all tumor cells and was defined as (tumor subset + 0.5)/(all tumor + 1). The IT index describes the abundance of a given immune or stromal subset relative to tumor cells and was defined as (immune or stromal subset + 0.5)/(immune or stromal subset + all tumor + 1). Laplace smoothing was applied by adding 0.5 to the subset count and 1 to denominators to prevent index values of exactly 0 or 1. All indices were then analyzed on the logit scale [50,51].

BRS molecular subtypes (BRS1–3) were obtained from de Jong et al. BRS1 and BRS2 were combined a priori to increase statistical power for comparison with BRS3. Indices were compared between BCG responders and nonresponders and across BRS subtypes. Group comparisons were made using two-sided Wilcoxon tests, and we report the Hodges–Lehmann (HL) median difference with 95% confidence intervals (CI) [52,53,54]. For interpretability, logit-transformed values were back-transformed using the inverse logit and are reported as percentages.

Categorical variables included age, sex, smoking status, tumor focality, concomitant CIS, LVI, histological variant, T1 substage, and EAU risk group. Age and tumor size were dichotomized at the cohort median and 3 cm cutoff, respectively. Comparisons of categorical variables across BCG response or BRS subtype were performed using Fisher’s exact test. Survival analyses employed RFS and PFS as endpoints. Logit-transformed clusters were treated as continuous variables in Cox proportional hazard models; for Kaplan–Meier plots, clusters were dichotomized at the cohort median. Dichotomization was used only for Kaplan–Meier visualization and associated log-rank tests, whereas hazard ratios (HRs) with 95% CIs were estimated using univariable and multivariable Cox proportional hazards models.

CNs were defined as clusters of cells with similar mean marker expression in their nearest neighbors. SCs were defined as clusters of CN compositions describing how different CNs are arranged around each cell in a larger spatial context. For analysis, CNs and SCs were encoded as binary variables (present or absent) for each patient. Associations with BCG response and BRS subtype were tested using Fisher’s exact test. Associations with PFS and RFS were assessed using univariable Cox models, and survival differences were visualized using Kaplan–Meier curves. Pairwise interaction analysis was performed to identify enriched contacts between clusters. Interaction analysis detects whether two cell types appear next to each other more often than expected by chance. Directed interactions were collapsed into undirected pairs, deduplicated, and restricted to interactions occurring in at least 60% of cases. These interaction pairs were then tested for associations with BCG response, BRS subtype, PFS, and RFS using Fisher’s exact tests or Cox models, respectively. All multiple comparisons were corrected using the false discovery rate (FDR).

Given the limited number of progression and recurrence events, Cox models were interpreted as exploratory effect-size estimates, and internal validation procedures (e.g., bootstrap correction) were not performed. Only associations that remained significant after FDR correction are reported as statistically robust; all other findings are presented as exploratory.

## 3. Results

### 3.1. Spatial Single-Cell IMC Analysis

In the single-cell IMC analysis, a total of 530,728 cells were segmented across 82 TMA cores. Unsupervised clustering initially identified 20 phenotypic populations. Highly similar clusters were merged to yield 18 final clusters. Based on dominant marker expression, clusters were annotated as 9 immune clusters (46% of all segmented cells), 2 stromal clusters (12%), 6 tumor clusters (41%), and 1 undefined cluster (1%). Immune clusters comprised CD20^+^ B cells, CD4^+^ and CD8^+^ T cells, CD38^+^ plasma cells, CD163^+^ CD204^+^ M2 macrophages, CD45^+^ CD11b^+^ HLA-DR^−^ eMDSC-like myeloid cells, and CD15^+^ granulocytes. Ki67^+^ immune cells formed a distinct proliferative immune cluster. One immune cell cluster located within the tumor compartment (ICTC) was defined by spatial position rather than lineage (Figure S2).

**Figure 4:**
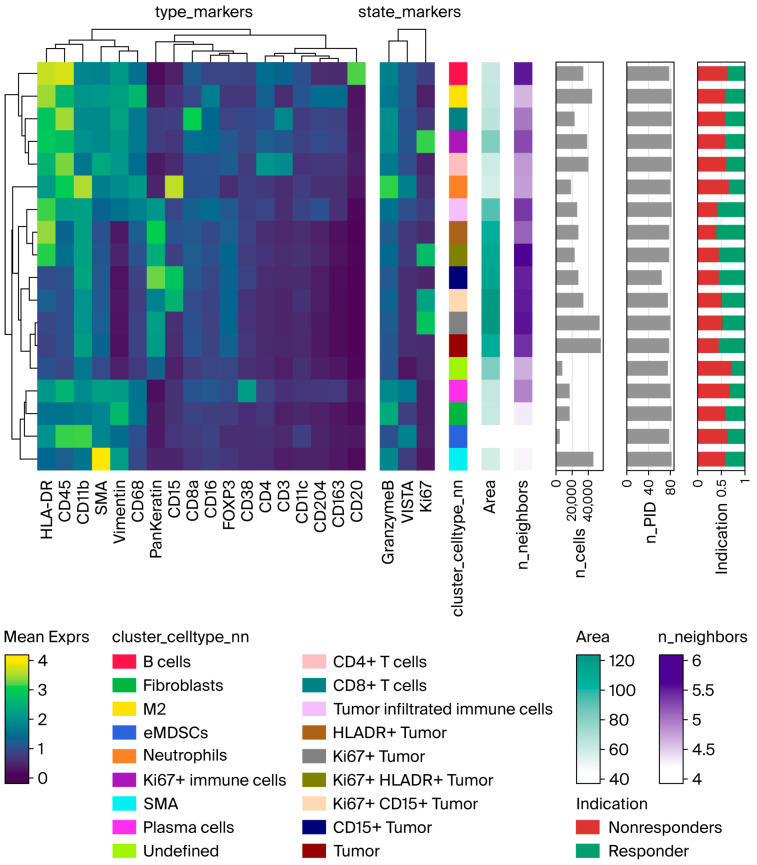
Cluster-level marker expression and distribution metrics visualized by IMC. Heatmap showing mean expression of lineage-associated type markers and functional state markers across clusters, ordered by hierarchical similarity. Annotated bars indicate cluster identity, relative spatial area, mean number of neighboring cell interactions (n_neighbors), total segmented cells (n_cells), inter-patient occurrence (n_PID), and clinical response distribution (indication, responder vs. nonresponder). Color scales represent normalized marker intensity, area contribution, and interaction density. Abbreviations: ICTC, immune cell cluster located within the tumor compartment; eMDSCs, early myeloid-derived suppressor cells; HLADR, human leukocyte antigen DR.

Markers most important for defining phenotypic clusters included HLA-DR, CD45, CD11b, SMA, vimentin, CD68, pan-keratin, CD15, CD8a, CD16, FOXP3, CD38, CD4, CD3, CD11c, CD204, CD163, and CD20. Functional state markers Ki67 and VISTA were included to capture proliferative and immunoregulatory states. Ki67 expression was elevated in the proliferative immune cluster and in the Ki67^+^ tumor subsets. VISTA expression was observed in granulocyte, plasma cell, and eMDSC-like clusters (Figure 4).

#### 3.1.1. Clinical Characteristics Associated with BCG Treatment Response

Among 82 patients with high-risk NMIBC, 45 (55%) experienced BCG failure, and 37 (45%) responded. BRS subtype information was available for 79 patients, of whom 67% were classified as BRS1/2 and 33% as BRS3. BCG failure occurred more frequently in BRS3 than in BRS1/2 (69% vs. 45%). Baseline clinical characteristics, including age, sex, smoking status, tumor focality, CIS, LVI, histological variant, T1 substage, and EAU risk group, were similarly distributed between BCG nonresponder and responder groups. Tumor size was unreported in 66% of cases (Table 4).

#### 3.1.2. Immune, Stromal, and Tumor Cell Clusters Associated with BCG Treatment Response

**Figure 5:**
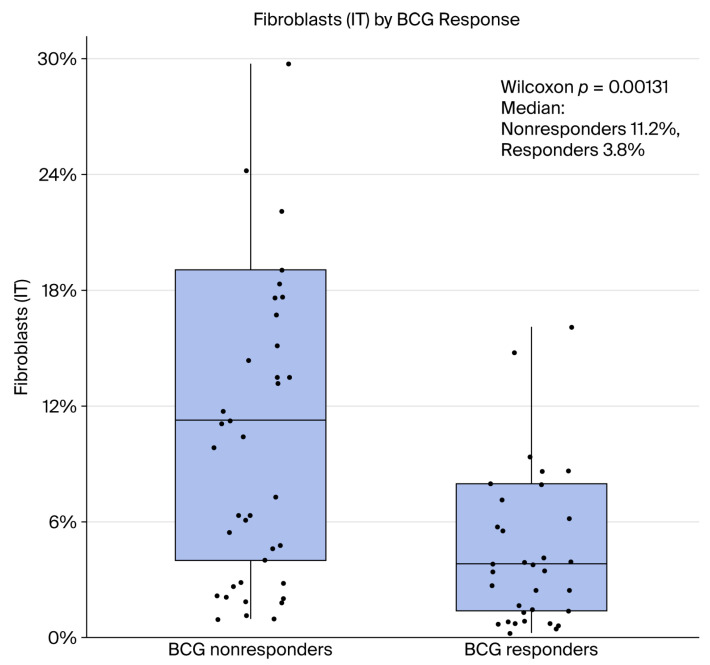
Fibroblast fractions (IT index) between BCG nonresponders (*n* = 45) and BCG responders (*n* = 37). Abbreviations: IT index, relative abundance of immune and stromal clusters normalized to tumor clusters.

In the cohort of 82 NMIBC patients, we assessed differences in cluster abundance between BCG response groups using the TT (tumor composition), II (immune composition), and IT (immune/stromal abundance relative to tumor) indices. ICTC clusters (II) were more prevalent in BCG responders, whereas plasma cell clusters (II) were higher in BCG nonresponders, though neither association remained significant after FDR correction. In the IT index, fibroblasts were significantly enriched in BCG nonresponders and remained significant even after FDR adjustment. Other clusters, including α-SMA^+^ stromal cells, plasma cells, and M2 macrophages, were also more abundant in nonresponders but did not remain significant after correction. No clusters in the TT index showed significant differences (Table 5 and Figure 5).

#### 3.1.3. Immune, Stromal, and Tumor Cell Clusters Associated with BRS Subtypes

We compared TT, II, and IT indices across BRS subtypes in the 79 patients with available BRS annotation. No clusters within the II or TT indices differed significantly between BRS groups. Within the IT index, several clusters, including Ki67^+^ immune cells, ICTC, plasma cells, granulocytes, CD8^+^ T cells, M2 macrophages, CD4^+^ T cells, and fibroblasts, were more abundant in BRS3 than in BRS1/BRS2. However, none of these differences remained statistically significant after FDR correction (Table 6).

#### 3.1.4. Univariable Survival Analysis of Clinical Characteristics and BRS Subtypes

Time-to-event data were available for 79 of the 82 patients. Stage progression occurred in 28 (35%) patients and tumor recurrence in 42 (53%) patients. LVI and multifocality were significantly associated with shorter PFS (HR 4.24; *p* < 0.001 and HR 2.37; *p* = 0.038, respectively). A significant difference in PFS between sexes was observed in the log-rank test (*p* = 0.012), but Cox regression could not be performed because no progression events occurred in female patients. No other clinical variables were associated with PFS or RFS. The BRS3 subtype showed higher progression risk (HR 1.96; *p* = 0.077) and recurrence risk (HR 1.66; *p* = 0.104), although neither association was statistically significant (Table 7).

#### 3.1.5. Univariable Survival Analysis of Immune, Stromal, and Tumor Cell Clusters

**Figure 6:**
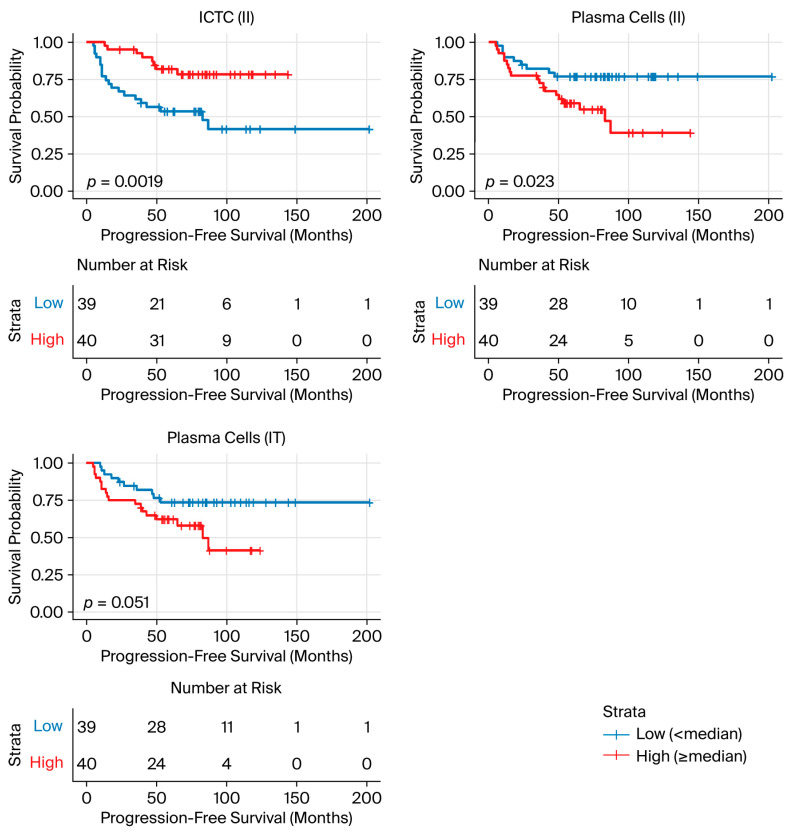
Kaplan–Meier curves showing progression-free survival for the ICTC (II), plasma cell (II), and plasma cell (IT) clusters. Median values used for dichotomization were 8.87%, 4.19%, and 5.62%, respectively. Log-rank *p*-values are shown in each panel. Abbreviations: ICTC, immune cell cluster located within the tumor compartment; II index, relative composition of immune clusters among all immune clusters; IT index, relative abundance of immune and stromal clusters normalized to tumor clusters.

Univariable survival analyses were performed for immune, stromal, and tumor cell clusters using the II, TT, and IT indices. A total of 79 patients with available survival data were included in the Cox regression and Kaplan–Meier analyses. In univariable Cox models for PFS, the ICTC cluster (II) was associated with lower progression risk, whereas plasma cell clusters (II and IT) were associated with higher risk. After FDR correction of Cox *p*-values, the ICTC cluster (II), plasma cell clusters (II), and plasma cell clusters (IT) remained significant (FDR < 0.05) (Table 8 and Figure 6). No stromal or tumor clusters were statistically significant in the univariable Cox analyses.

For RFS analysis, the ICTC cluster (II) was associated with better RFS, whereas fibroblast (IT), α-SMA^+^ stromal (IT), and plasma cell clusters (IT) were associated with worse RFS. However, none of these associations remained significant after FDR correction (Table 9).

#### 3.1.6. Multivariable Survival Analysis of Immune, Stromal, and Tumor Cell Clusters

In multivariable Cox models adjusted for all clinical variables, the ICTC cluster (II), plasma cell cluster (II), and plasma cell cluster (IT) remained independently associated with progression after FDR correction. Plasma cells (II) showed the strongest association (HR 2.28; 95% CI 1.37–3.79; *p* = 0.0015; FDR = 0.004), followed by plasma cells (IT) (HR 1.25; 95% CI 1.06–1.48; *p* = 0.0076; FDR = 0.012). The ICTC cluster (II) remained protective (HR 0.67; 95% CI 0.49–0.92; *p* = 0.012; FDR = 0.013). For recurrence, the fibroblast (IT) fraction was the only stromal feature that remained statistically significant after FDR correction (HR 1.26; 95% CI 1.06–1.50; *p* = 0.009; FDR = 0.038). The ICTC cluster (II) was associated with a lower recurrence risk (HR 0.74; 95% CI 0.57–0.96; *p* = 0.024; FDR = 0.048), although this effect was weaker.

#### 3.1.7. Cellular Neighborhood (CN) Analysis

CNs, defined as clusters of cells with similar mean marker expression in their nearest neighbors, were evaluated. We identified 10 CNs (CN1–CN10). CN1 and CN4 were the most prevalent, each observed in 96% of patients, followed by CN2 (89%) and CN8 (88%). CN1 was enriched for the α-SMA^+^ cluster, whereas CN4 was dominated by granulocytes, M2 macrophages, eMDSC-like cells, and fibroblast clusters. CN2 consisted of tumor and Ki-67^+^ tumor clusters, and CN8 comprised the CD4^+^ T-cell cluster together with the plasma cell cluster (Figure S3). The presence of CN2 was associated with a reduced risk of progression in the Cox model (HR 0.34, 95% CI 0.13–0.91, *p* = 0.032). However, this association did not remain statistically significant after FDR correction. We did not find any significant associations between CNs and BCG response or BRS subtype.

#### 3.1.8. Spatial Context (SC) Analysis

**Figure 7:**
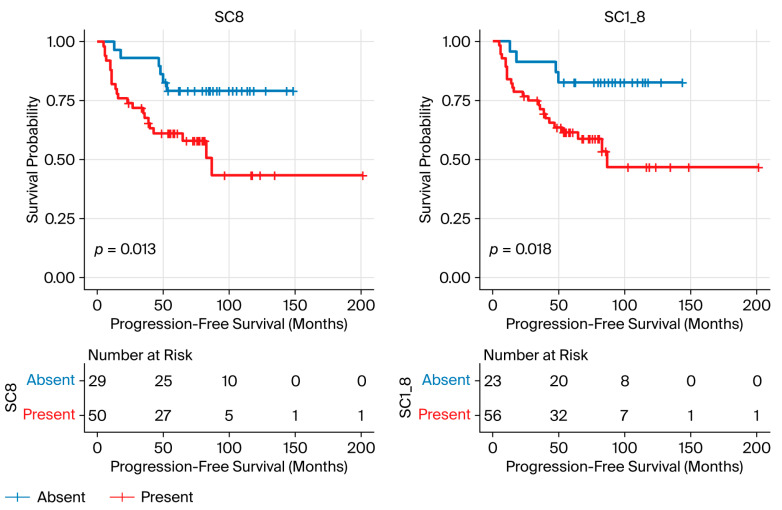
Kaplan–Meier curves with risk tables showing progression-free survival stratified by the presence or absence of the spatial contexts SC8 and SC1_8. The CD4^+^ T-cell and plasma cell aggregates observed around blood vessels most likely represent perivascular immune infiltrates. Abbreviations: SC, spatial context; SC8, a spatial context enriched for CN8 (α-SMA^+^ cluster); SC1_8, a spatial context enriched for CN1_8 (α-SMA^+^, CD4^+^ T-cell, and plasma cell clusters).

SCs, defined as recurring combinations of CNs, were examined. A total of 129 SCs were identified. The most prevalent SCs across patients were CN1_4 (78%), CN1 (77%), and CN1_8 (72%). SC8, enriched for CN8 (α-SMA^+^ cluster), and SC1_8, enriched for CN1_8 (α-SMA^+^, CD4^+^ T-cell, and plasma cell clusters), were associated with shorter PFS in the Cox model (HR 3.00, 95% CI 1.20–7.45, *p* = 0.018; FDR = 0.10, and HR 3.38, 95% CI 1.16–9.82, *p* = 0.025; FDR = 0.10, respectively) (Figure 7). No significant associations were found between SCs and BCG response or BRS subtype.

#### 3.1.9. Interaction Analysis

Interaction analysis, which identifies cluster pairs that occur as direct neighbors more often than expected by chance, was performed. The CD4^+^ T cell–Ki67^+^ immune cluster pair was associated with improved BCG response (OR 0.25, 95% CI 0.05–0.89; *p* = 0.019; FDR = 0.42). The α-SMA^+^–M2 macrophage pair (OR 0.24, 95% CI 0.05–0.96; *p* = 0.024; FDR = 0.27) and the CD4^+^ T cell–fibroblast pair (OR 0.31, 95% CI 0.10–0.92; *p* = 0.024; FDR = 0.27) were less frequent in BRS3 than in BRS1/2. For RFS, the CD4^+^ T cell–Ki67^+^ immune pair (HR 0.40, 95% CI 0.21–0.76; *p* = 0.006; FDR = 0.12) and the CD8^+^ T cell–Ki67^+^ immune pair (HR 0.49, 95% CI 0.26–0.90; *p* = 0.022; FDR = 0.24) were associated with longer RFS. None of these associations remained significant after FDR correction (Figure S4 and Table S1).

### 3.2. Performance of the IMC-GA-MIL Model for BCG Response Classification

#### 3.2.1. Classification Performance

The IMC-GA-MIL model demonstrated strong predictive performance on the independent patient-level hold-out cohort (n = 10; 5 responders/5 non-responders). Using a decision threshold of 0.5, the model correctly classified all five BCG non-responders and four of five responders (true positives = 4, true negatives = 5, false positives = 0, false negatives = 1), corresponding to 90% accuracy, precision = 1.00, recall = 0.80, and an F1-score of 0.89 (Figure S5). The model produced continuous probability estimates ranging from 0 to 1, with non-responders consistently assigned low predicted scores (≤0.18), whereas responders received higher scores (0.53–0.82), aside from one responder with an intermediate score of 0.19 (Table S2). This complete rank separation between classes resulted in ROC–AUC = 1.00 and PR–AUC = 1.00, reflecting that all 25 positive–negative patient pairs were correctly rank-ordered. An exact 95% (Clopper–Pearson) binomial CI for the AUC, computed from the 25 pairwise positive–negative comparisons, was 0.86–1.00, demonstrating the discrete and inherently uncertain nature of AUC estimation in such a small cohort. The model further achieved a Brier score of 0.104, indicating good overall probabilistic calibration given the limited cohort size.

A 3-fold patient-level cross-validation performed during stage-two MIL training (with the feature extractor frozen) showed stable and consistently decreasing training loss across folds, supporting the robustness of the optimization procedure (Figure S6). Finally, when applied to the six IMC images excluded from GA-MIL training, the model correctly classified all cases, suggesting stable generalization to patients not involved in MIL model fitting.

#### 3.2.2. Channel Sensitivity Analysis

We assessed sensitivity to individual channels using a leave-one-channel-out perturbation strategy. Each channel was removed in turn by setting its values to zero, while the remaining channels were kept unchanged, as the model architecture requires a fixed 18-channel input. Models were not retrained for this analysis; performance changes, therefore, reflect post hoc sensitivity to individual channels rather than adaptation during training. The largest negative changes in patch-level classification performance were observed following the removal of CD14, FOXP3, CD68, CD8, and CD11b. These channels also had the strongest impact on GA-MIL performance. Removal of FOXP3 or CD8 reduced performance to 7 of 10 correctly classified images, whereas removal of CD68 or CD11b reduced performance to 6 of 10, indicating that these markers are particularly important for patient-level prediction (Table S3).

#### 3.2.3. Attention Analysis

**Figure 8:**
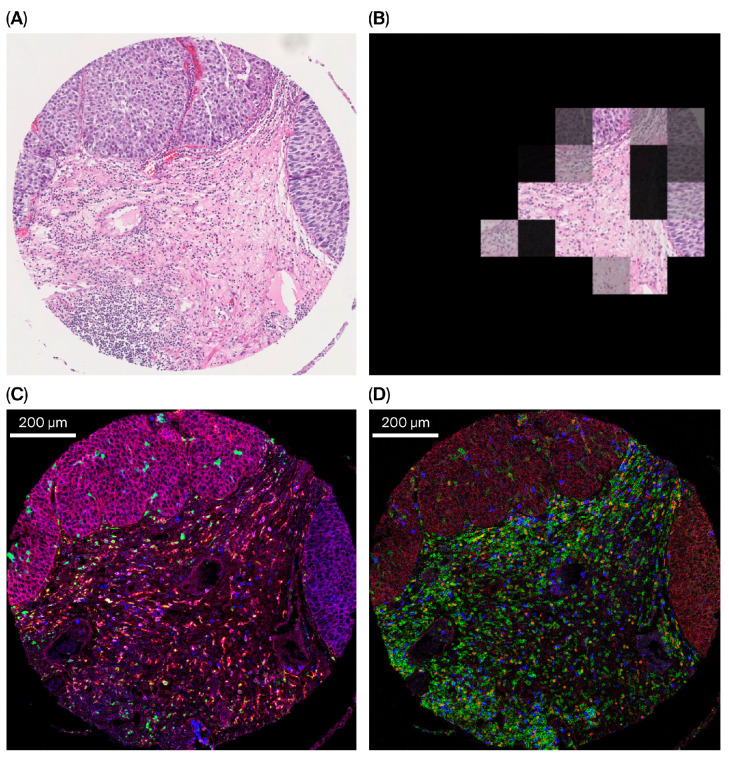
The IMC-GA-MIL model assigns attention scores to image patches, indicating their contribution to the final image-level prediction of BCG response. (**A**) H&E-stained TMA section (P41). (**B**) Attention-weighted patches overlaid on the H&E image; overlaid regions indicate patches with higher attention scores contributing to the patient-level prediction. (**C**) Corresponding IMC image showing myeloid marker expression with CD14 (red), CD11b (blue), and CD68 (green). Co-expression of CD14 and CD11b appears purple, consistent with MDSC-like patterns, and co-expression of CD14 and CD68 appears yellow, consistent with macrophage-associated patterns. (**D**) IMC image showing T-cell marker expression with CD4 (green) and FOXP3 (red); co-expression identifies CD4^+^ FOXP3^+^ T cells and CD8 (blue). Scale bars, 200 µm.

The attention scores generated by the GA-MIL method show which patches contributed most to the final image-level prediction. Representative attention maps overlaid on H&E-stained TMA sections adjacent to the corresponding IMC images are shown in Figure 8.

## 4. Discussion

In NMIBC, an immunosuppressive TME has been linked to poor prognosis and treatment failure [55]. A recent multicenter study by de Jong et al. identified three molecular subtypes (BRS1–3) in high-risk NMIBC using whole-transcriptome sequencing. BRS1 and BRS2 were associated with favorable outcomes. BRS3 was characterized by an immunosuppressive phenotype with infiltration of B cells, Tregs, M2 macrophages, and exhausted CD8^+^ T cells, and it was an independent predictor of progression [8]. In the present study, using tumor samples from the same cohort, we confirmed that BRS3-like tumors were enriched for plasma cells, fibroblasts, granulocytes, CD4^+^ and CD8^+^ T cells, and M2 macrophages, consistent with an immune-rich but functionally suppressed TME. In line with these findings, other studies have reported that a high neutrophil-to-lymphocyte ratio, increased M2 macrophages, and a low T cell-to-MDSC ratio are linked to poor BCG response in bladder cancer [16,17,20,56]. A Th1-dominant adaptive immune response supports effective BCG therapy, whereas Th2 polarization is associated with poor prognosis [25]. BCG nonresponders have also been reported to show increased infiltration of exhausted CD8^+^ PD-1^+^ T cells and Tregs [23,25]. VISTA (PD-1H), an immune checkpoint molecule primarily expressed on hematopoietic cells, including dendritic cells, granulocytes, macrophages, and monocytes, transmits suppressive signals to T cells and contributes to T cell exhaustion [57]. In our study, VISTA was highly expressed in eMDSC-like cells, plasma cells, and granulocytes, consistent with an immunosuppressive TME and supporting the BRS3-like phenotype. High VISTA expression has also been associated with higher stage, grade, and recurrence rates in bladder cancer [58].

We also found that BCG nonresponse was associated with a fibroblast- and plasma cell–rich microenvironment, whereas intratumoral accumulation of immune cells (ICTCs) was linked to better prognosis. In healthy tissue, fibroblasts maintain tissue integrity and contribute to homeostasis. In the TME, they may transform into CAFs that modify the extracellular matrix, secrete signaling molecules, and influence immune cell activity. CAFs are heterogeneous in both phenotype and function. α-SMA^+^ myofibroblast-like CAFs deposit collagen-rich extracellular matrix, increasing stromal density and stiffness. This fibrotic barrier can support tumor growth and restrict immune cell infiltration. By contrast, vimentin^+^ α-SMA^−^ CAFs primarily regulate immune activity. They produce signaling molecules and cytokines that alter immune cell behavior and can suppress immune activation [30,59,60,61]. In line with our findings, Feng et al. identified fibroblast-rich regions associated with poor prognosis and abnormal vasculature in MIBC [34]. Activated vimentin^+^ α-SMA^−^ CAFs often express fibroblast activation protein, and their presence has been linked to stage progression in BCG-treated patients with HG T1 NMIBC [62]. Plasma cells are key components of humoral immunity but can exert dual effects in the TME. In bladder cancer, IgG1-producing plasma cells have been associated with antitumor activity, whereas IgA1-dominant populations may contribute to tumor-promoting inflammation [63,64]. In our study, CD38^+^ plasma cells were the strongest independent predictor of BCG failure. CD38 is an enzyme that breaks down nicotinamide adenine dinucleotide into intermediates that are further converted to adenosine. Adenosine can function as an immunosuppressive signal by binding to receptors on T and NK cells, inhibiting their activity [65,66]. A pilot study in MIBC similarly found that tumors with high CD38^+^ immune cells had an immune-refractory phenotype and that treatment with the anti-CD38 monoclonal antibody daratumumab reduced immunosuppressive populations in the TME [67].

Tumor cells use the vasculature to support their growth and metastasis. In our SC analysis, α-SMA^+^ vasculature co-occurring with CD4^+^ Th2 cells and plasma cells was associated with shorter PFS. Consistent with this observation, perivascular stromal cells have been shown to promote type 2 immunity and contribute to an immunosuppressive microenvironment [68]. Similar perivascular niches have been identified as hubs of immune evasion and tumor progression across multiple cancers [69]. In our interaction analysis, several interaction pairs were associated with BCG response and survival in unadjusted analyses, but none remained significant after multiple-testing correction. Therefore, these findings should be interpreted cautiously.

Following BCG therapy, neutrophils, macrophages, and dendritic cells are among the first immune cells recruited to the bladder wall [12]. These cells recognize BCG through Toll-like receptors and the co-receptor CD14, which is mainly expressed on myeloid cells and triggers inflammatory signaling [70,71]. In our analysis, CD14 was highly expressed in both immune and tumor cells. In the IMC-GA-MIL model, removal of CD14 resulted in the largest reduction in predictive performance, followed by FOXP3, CD8, CD11b, and CD68. Therefore, both myeloid markers (CD14, CD11b, CD68) and T cell markers (CD8, FOXP3) appear to contribute substantially to model performance. This pattern is consistent with the BCG failure phenotype, characterized by myeloid activity and Treg involvement. Using IMC, Pour et al. similarly identified a CD14^+^ CD11b^+^ population at the tumor–stroma interface associated with poor BCG response. Similar to the proposed IMC-GA-MIL approach, their analysis did not require single-cell delineation but instead relied on image-level marker expression patterns [72]. Furthermore, GA-MIL attention maps were used to visually examine which tissue regions (patches) contributed to the model predictions. In BCG nonresponders, high-attention regions were observed in areas enriched for immunosuppressive immune markers. These observations were based on visual inspection and were not quantitatively assessed.

Furthermore, we observed that high ICTCs, mainly cytotoxic T cells, were associated with better prognosis. This finding is consistent with prior reports showing that intraepithelial CD8^+^ T cells predict a favorable outcome in bladder cancer [73].

BCG has been studied for decades, but its mechanisms remain unclear, limiting the identification of biomarkers for targeted therapy. Another challenge is the lack of large, prospective, well-annotated cohorts of BCG-treated patients with matched specimens (pre- and post-BCG), standardized treatment protocols, and consistent follow-up. Existing biomarker studies are often small, retrospective, and heterogeneous in endpoints and methods, which makes comparison across studies difficult [74]. Our study addressed several of these challenges. We used a well-defined multicenter cohort with standardized clinical data and clear EAU-based definitions of BCG response. Building on the transcriptomic findings reported by de Jong et al., we applied IMC to examine these mechanisms at single-cell resolution. We confirmed that immunosuppressive cell phenotypes were associated with poor BCG response, supporting early consideration of immune checkpoint blockade or timely cystectomy. Unfortunately, in our analysis of IC markers, PD-1, PD-L1, PD-L2, and LAG-3 antibodies showed limited or undetectable staining. By contrast, strong intratumoral T-cell infiltration may represent a biomarker of durable BCG response.

Our cohort consisted exclusively of high-risk T1 NMIBC, and clinicopathological variables were broadly comparable between response groups; the key IMC-derived features (ICTC, plasma cells, fibroblasts) also remained associated with outcome after adjustment for available clinical covariates.

Our study has several limitations. Although several antibodies in our IMC panel were validated by conventional IHC during panel optimization, we did not perform systematic orthogonal validation of the IMC-derived phenotypes across the full cohort. In particular, the enrichment of fibroblasts and CD38^+^ plasma cells was not confirmed using independent methods such as IHC or multiplex immunofluorescence. These biological findings should therefore be interpreted as exploratory, and future work will incorporate targeted orthogonal validation to confirm these cell-state and spatial signatures.

The sample size was relatively small, limiting statistical power. Moreover, analyses were performed on TMAs rather than whole-slide images. Although TMAs are efficient and standardized, they sample only a small fraction of the tumor and may miss intratumoral heterogeneity. Pre-analytical factors may also have influenced staining quality. Sections were stored at –80 °C for approximately one year before staining, which may have reduced antigen preservation and antibody performance for unstable markers. Consistent pre-analytical handling and rigorous quality control are essential in future studies. Visual quality checks were performed at each processing step to maintain data quality and minimize technical artifacts. However, manual cluster annotation and parameter selection involve subjective judgment. Our findings should be validated in larger prospective cohorts to confirm the predictive and clinical value of the spatial biomarkers. Furthermore, the IMC-GA-MIL framework was constrained by the limited amount of available training data. To mitigate this, we used transfer learning with a small backbone model (ResNet-18, adapted for 18 channels), pretrained on ImageNet. We first learned the feature embedder using a patch-based framework, followed by training of the aggregator and final classifier in the GA-MIL setup with a frozen backbone. With larger datasets, this framework could be trained end-to-end, which may be more favorable, and alternative feature embedding architectures could be tested. The channel sensitivity analysis could also be expanded by retraining the network using different combinations of channel groups. Although the IMC-GA-MIL model showed promising discriminative ability, these findings must be interpreted as exploratory because the independent test cohort was very small. Performance estimates from such limited data are inherently unstable, and external validation in larger, independent IMC cohorts will be essential before any clinical conclusions can be drawn.

## 5. Conclusions

BCG failure in high-risk NMIBC was associated with an immunosuppressive tumor microenvironment, consistent with the BRS3 phenotype described by de Jong et al. In multivariable analyses of spatial single-cell IMC features, CD38^+^ plasma cell abundance, measured both within the immune compartment and relative to tumor cells, was independently associated with disease progression, whereas immune cells localized within the tumor compartment were independently protective. By contrast, fibroblast abundance relative to tumor cells was the only stromal feature independently associated with tumor recurrence. In this cohort, the IMC-GA-MIL model demonstrated predictive potential for BCG response. Myeloid- and T-cell-associated signal patterns involving CD14, CD11b, CD68, CD8, and FOXP3 contributed most strongly to the prediction, consistent with the myeloid and T-cell context observed in the spatial single-cell analyses.

## Figures and Tables

**Table 1:** Overview of patient data included for single-cell IMC and IMC-GA-MIL analyses.

Image Analysis	Number of Patients
Patients diagnosed with high-risk NMIBC	427
Selected for single-cell IMC analysis	88
- Excluded because of poor staining quality	6
Included in BCG response analysis	82
- Missing survival and BRS subtype data	3
Included in PFS analysis	79
Included in RFS analysis	79
Included in BRS subtype association analysis	79
Selected for IMC-GA-MIL analysis (highest quality images)	69
- Included in the training set	59
- Included in the test set	10

Abbreviations: IMC, imaging mass cytometry; IMC-GA-MIL, IMC-specific, gated attention-based multiple instance learning; RFS, recurrence-free survival; PFS, progression-free survival; BRS, BCG response subtype classification, as defined by de Jong et al.

**Table 2:** Antibody marker panel used for IMC analysis.

Name	Marker	Cell Type	Ab cc. ^1^	Clone	Product Nr.
Dy161	CD20	B cells	1:400	H1	3161029D
Dy162	CD8a	T cytotoxic	1:100	C8/144B	3162034D
Dy163	CD11b	MDSCs	1:100	EPR1344	91H007163
Er166	CD56 ^2^	NK cells	1:75	E7X9M	88856SF
Er167	Granzyme B	Cytotoxic cells	1:100	EPR20129-217	3167021D
Er168	Ki67	Proliferation	1:50	B56	3168022D
Er170	CD3	T cells	1:100	Polyclonal	3170019D
Eu151	Vimentin	Stroma cells	1:300	D21H3	91H002151
Gd155	FOXP3	Tregs	1:50	PCH101	3155018D
Gd156	CD4	Th cells	1:200	EPR6855	3156033D
Gd160	VISTA	Immune checkpoint	1:50	D1L2G	3160025D
Ho165	PD-1	Immune checkpoint	1:50	EPR4877(2)	3165039D
Lu175	CD25	Tregs	1:50	EPR6452	3175036D
Nd142	Pan-keratin	Epithelial cells	1:200	C11	91H014142
Nd143	CD38 ^3^	Plasma cells	1:50	EPR4106	ab108403
Nd144	CD14	MDSCs	1:100	EPR3653	3144025D
Nd145	T-bet	Th1 cells	1:50	D6N8B	3145015D
Nd146	CD16	Neutrophils	1:50	EPR16784	3146020D
Nd148	CD278-ICOS	Immune checkpoint	1:50	D1K2T	3148021D
Pr141	SMA	SMA	1:200	1A4	3141017D
Sm147	CD163	M2 macrophages	1:100	EDHu-1	3147021D
Sm149	CD15	MDSCs	1:50	W6D3	3149026D
Sm152	CD45	Lymphocytes	1:100	CD45-2B11	3152018D
Sm154	CD11c	Dendritic cells	1:100	EP1347Y	3999999-5 + clone
Tb159	CD68	Macrophages	1:50	KP1	3159035D
Tm169	GATA3 ^2^	Th2 cells	1:50	D13C9	5852BF
Yb171	TGF-β ^2^	Tregs	1:250	OTI4F11	CF809351
Yb174	HLA-DR	MHC II	1:200	LN3	3174025D
Yb176	CD204	M2 macrophages	1:75	OTI8C11	CF8027781
Ir191	DNA	Nuclear	1:400	–	–
Ir193	DNA	Nuclear	1:400	–	–

^1^ Ab cc.: antibody concentration; Suppliers: ^2^ Cell signaling technology; ^3^ Abcam; all others were from Standard BioTools™. Abbreviations: MDSCs, Myeloid-derived suppressor cells; Tregs, Regulatory T cells; Th cells, T helper cells; SMA, Smooth Muscle Actin; NK cells, Natural Killer cells; MHC II, Major Histocompatibility Complex class II.

**Table 3:** Antibody marker panel used for prediction of BCG response in the IMC-GA-MIL model.

Category	Markers
Structural markers	Pan-keratin, Vimentin
Immune cell markers	CD11b, CD14, CD15, CD163, CD20, CD204, CD4, CD56, CD68, CD8
Transcription factors	T-bet, FOXP3
Immune checkpoint	CD278-ICOS, VISTA
Functional immune markers	Granzyme B, TGF-β

**Table 4:** Summary of clinical characteristics associated with BCG treatment response.

Clinical Characteristic		Overall	BCG Responder	BCG Nonresponder	*p* ^2^
		N = 82 ^1^	n = 37 ^1^	n = 45 ^1^	
Age	<69.5	41 (50%)	17 (46%)	24 (53%)	0.657
	≥69.5	41 (50%)	20 (54%)	21 (47%)	
Sex	Female	12 (15%)	7 (19%)	5 (11%)	0.236
	Male	67 (82%)	30 (81%)	37 (82%)	
	Missing	3 (3.7%)	0 (0%)	3 (6.7%)	
Smoking	No	23 (28%)	10 (27%)	13 (29%)	0.764
	Yes	50 (61%)	24 (65%)	26 (58%)	
	Missing	9 (11%)	3 (8.1%)	6 (13%)	
Focality	Unifocal	37 (45%)	21 (57%)	16 (36%)	0.080
	Multifocal	43 (52%)	16 (43%)	27 (60%)	
	Missing	2 (2.4%)	0 (0%)	2 (4.4%)	
Size	<3 cm	17 (21%)	10 (27%)	7 (16%)	0.116
	>3 cm	11 (13%)	7 (19%)	4 (8.9%)	
	Missing	54 (66%)	20 (54%)	34 (76%)	
CIS	No	63 (77%)	29 (78%)	34 (76%)	0.799
	Yes	19 (23%)	8 (22%)	11 (24%)	
LVI	No	72 (88%)	36 (97%)	36 (80%)	0.058
	Yes	7 (8.5%)	1 (2.7%)	6 (13%)	
	Missing	3 (3.7%)	0 (0%)	3 (6.7%)	
Histological variant	UCC	71 (87%)	34 (92%)	37 (82%)	0.301
	Others	8 (9.8%)	3 (8%)	5 (11%)	
	Missing	3 (3.7%)	0 (0%)	3 (6.7%)	
T1 substage	T1 micro	19 (23%)	11 (30%)	8 (18%)	0.201
	T1 extensive	60 (73%)	26 (70%)	34 (76%)	
	Missing	3 (3.7%)	0 (0%)	3 (6.7%)	
EAU risk group	High-risk	28 (34%)	14 (38%)	14 (31%)	0.641
	Very high-risk	54 (66%)	23 (62%)	31 (69%)	
BRS subtype	BRS1/2	53 (65%)	29 (78%)	24 (53%)	0.057
	BRS3	26 (32%)	8 (22%)	18 (40%)	
	Missing	3 (3.7%)	0 (0%)	3 (6.7%)	

^1^ Values are presented as n (%). ^2^ *p*-values were calculated using Fisher’s exact test. Abbreviations: LVI, lymphovascular invasion; CIS, carcinoma in situ; EAU, European Association of Urology risk group; BRS, BCG response subtype.

**Table 5:** Immune, stromal, and tumor cell clusters associated with BCG treatment response.

Cluster	BCG Nonresponder Median % (95% CI)	BCG Responder Median % (95% CI)	HL (Logit) (95% CI)	*p*	FDR
ICTC (II)	6.06 (4.59, 9.67)	14.41 (8.04, 22.02)	–0.71 (–1.32, –0.12)	0.019	0.127
Plasma cells (II)	6.00 (4.04, 8.20)	3.84 (2.08, 6.04)	0.57 (0.01, 1.23)	0.046	0.172
Fibroblasts (IT)	11.21 (6.29, 15.10)	3.80 (2.43, 6.14)	1.13 (0.48, 1.80)	0.001	0.035
α-SMA^+^ (IT)	29.02 (17.56, 35.54)	10.99 (8.48, 16.99)	0.83 (0.24, 1.45)	0.008	0.107
Plasma cells (IT)	8.23 (5.12, 17.45)	4.28 (0.59, 9.75)	1.41 (0.20, 2.57)	0.017	0.127
M2 macrophages (IT)	18.76 (12.99, 37.18)	10.22 (5.87, 20.21)	0.75 (0.01, 1.59)	0.048	0.172

Abbreviations: HL, Hodges–Lehmann estimate of the median difference (logit scale) between BCG nonresponders and BCG responders, FDR, false discovery rate (Benjamini–Hochberg adjusted *p*-value); II index, relative composition of immune clusters among all immune clusters; IT index, relative abundance of immune and stromal clusters normalized to tumor clusters; ICTC, immune cell cluster located within the tumor compartment.

**Table 6:** Immune, stromal, and tumor cell clusters associated with BRS subtypes.

Cluster	BRS3 Median % (95% CI)	BRS1/2 Median % (95% CI)	HL (Logit) (95% CI)	*p*	FDR
Ki67^+^ immune cells (IT)	20.76 (7.70, 53.87)	7.65 (5.64, 14.90)	1.16 (0.20, 2.33)	0.015	0.109
Plasma cells (IT)	11.04 (5.28, 32.58)	4.24 (2.02, 8.23)	1.54 (0.25, 2.88)	0.016	0.109
ICTC (IT)	16.74 (8.47, 29.17)	6.88 (4.92, 11.00)	0.93 (0.22, 1.67)	0.015	0.109
Fibroblasts (IT)	13.31 (4.36, 29.72)	5.44 (2.80, 7.26)	1.05 (0.18, 2.07)	0.016	0.109
Granulocytes (IT)	14.20 (1.22, 33.66)	2.39 (1.12, 4.11)	1.53 (0.12, 2.78)	0.032	0.144
Cytotoxic T cells (IT)	22.54 (3.47, 47.16)	4.44 (3.49, 9.54)	1.48 (0.21, 2.71)	0.030	0.144
M2 macrophages (IT)	22.28 (10.41, 66.81)	12.22 (8.65, 18.91)	1.03 (0.04, 2.17)	0.044	0.150
CD4^+^ T cells (IT)	22.79 (9.82, 59.32)	9.97 (6.02, 15.74)	1.11 (0.01, 2.20)	0.049	0.150

Abbreviations: HL, Hodges–Lehmann estimate of the median difference (logit scale) between BRS3 and BRS1/2 groups; FDR, false discovery rate (Benjamini–Hochberg adjusted *p*-value); II index, relative composition of immune clusters among all immune clusters; IT index, relative abundance of immune and stromal clusters normalized to tumor clusters; ICTC, immune cell cluster located within the tumor compartment.

**Table 7:** Univariable survival analysis of clinical characteristics and BRS subtypes.

Clinical Parameters		Stage Progression				Tumor Recurrence			
		N Events (%)	*p* ^1^	HR 95% CI	*p* ^2^	N Events (%)	*p* ^1^	HR 95% CI	*p* ^2^
Age	<69	9/36 (25)	0.065	2.08 (0.94, 4.60)	0.071	17/36 (47)	0.362	1.33 (0.70, 2.69)	0.369
	≥69	19/43 (44)				25/43 (58)			
Sex	Male	28/67 (42)	0.012	Not estimable	-	37/67 (55)	0.627	1.25 (0.49, 3.18)	0.640
	Female	0/12 (0)				5/12 (42)			
Smoking	No	8/23 (35)	0.683	0.84 (0.36, 1.95)	0.682	13/23 (57)	0.407	0.75 (0.38, 1.46)	0.395
	Yes	17/50 (34)				26/50 (52)			
Focality	Unifocal	8/37 (22)	0.038	2.37 (1.03, 5.46)	0.043	16/37 (43)	0.153	1.58 (0.84, 2.98)	0.159
	Multifocal	18/40 (45)				24/40 (60)			
CIS	No	23/63 (37)	0.562	0.75 (0.29, 1.99)	0.567	34/63 (54)	0.877	0.94 (0.44, 2.03)	0.877
	Yes	5/16 (31)				8/16 (50)			
LVI	No	22/72 (31)	<0.001	4.24 (1.71, 10.5)	0.002	36/72 (50)	0.107	2.02 (0.85, 4.80)	0.113
	Yes	6/7 (86)				6/7 (86)			
Histological variant	UCC	26/71 (37)	0.579	0.67 (0.16, 2.83)	0.585	37/71 (52)	0.354	1.57 (0.62, 4.01)	0.344
	Other	2/8 (25)				5/8 (62)			
T1 substage	T1 micro	4/19 (21)	0.160	0.48 (0.17, 1.38)	0.172	8/19 (42)	0.412	0.72 (0.34, 1.57)	0.412
	T1 extensive	24/60 (40)				34/60 (57)			
EAU risk group	High-risk	8/28 (29)	0.311	1.53 (0.67, 3.48)	0.312	14/28 (50)	0.564	1.21 (0.63, 2.29)	0.568
	Very high-risk	20/51 (49)				28/51 (55)			
BRS subtype	1/2	15/53 (28)	0.070	1.96 (0.93, 4.12)	0.077	24/53 (45)	0.100	1.66 (0.90, 3.07)	0.104
	3	13/26 (50)				18/26 (69)			

^1^ *p*-value from Kaplan–Meier survival analysis (log-rank test); ^2^ *p*-value from Cox proportional hazards regression. Abbreviations: HR, hazard ratio; CI, confidence interval; LVI, lymphovascular invasion; CIS, carcinoma in situ; EAU, European Association of Urology; BRS, BCG response subtype.

**Table 8:** Univariable Cox proportional hazards analysis of immune, stromal, and tumor cell clusters for progression-free survival.

Stage Progression						
Cluster	Median (%)	N Events (%)	*p* ^1^ KM	HR (95% CI)	*p* ^2^ Cox	FDR
ICTC (II)	<8.87	20/39 (51%)	0.002	0.61 (0.47, 0.79)	<0.001	0.006
	≥8.87	8/40 (20%)				
Plasma cells (II)	<4.19	9/39 (23%)	0.023	1.82 (1.28, 2.60)	0.001	0.009
	≥4.19	19/40 (48%)				
Plasma cells (IT)	<5.62	10/39 (26%)	0.051	1.27 (1.10, 1.46)	0.001	0.009
	≥5.62	18/40 (45%)				
B cells (IT)	<2.92	10/39 (26%)	0.083	1.16 (1.03, 1.31)	0.013	0.090
	≥2.92	18/40 (45%)				
Granulocytes (IT)	<2.87	10/39 (26%)	0.057	1.16 (1.02, 1.32)	0.028	0.139
	≥2.87	18/40 (45%)				
CD4 T cells (IT)	<12.08	12/39 (31%)	0.326	1.18 (1.02, 1.36)	0.031	0.139
	≥12.08	16/40 (40%)				

^1^ *p*-value from Kaplan–Meier survival analysis (log-rank test); ^2^ *p*-value from Cox proportional hazards regression. Abbreviations: HR, hazard ratio; CI, confidence interval; FDR, false discovery rate (Benjamini–Hochberg adjusted *p*-value); II index, relative composition of immune clusters among all immune clusters; IT index, relative abundance of immune and stromal clusters normalized to tumor clusters; ICTC, immune cell cluster located within the tumor compartment.

**Table 9:** Univariable Cox proportional hazards analysis of immune, stromal, and tumor cell clusters for recurrence-free survival.

Tumor Recurrence						
Cluster	Median (%)	N Events (%)	*p* ^1^ KM	HR (95% CI)	*p* ^2^ Cox	FDR
ICTC (II)	<8.87	25/39 (64.10%)	0.036	0.75 (0.61, 0.92)	0.006	0.127
	≥8.87	17/40 (42.50%)				
Fibroblasts (IT)	<6.06	15/39 (38.46%)	0.012	1.18 (1.03, 1.36)	0.017	0.127
	≥6.06	27/40 (67.50%)				
α-SMA^+^ cells (IT)	<16.99	15/39 (38.46%)	0.014	1.20 (1.03, 1.40)	0.018	0.127
	≥16.99	27/40 (67.50%)				
Plasma cells (IT)	<5.62	17/39 (43.59%)	0.128	1.15 (1.02, 1.29)	0.019	0.127
	≥5.62	25/40 (62.50%)				

^1^ *p*-value from Kaplan–Meier survival analysis (log-rank test); ^2^ *p*-value from Cox proportional hazards regression. Abbreviations: HR, hazard ratio; CI, confidence interval; FDR, false discovery rate (Benjamini–Hochberg adjusted *p*-value); ICTC, immune cell cluster located within the tumor compartment; II index: relative composition of immune clusters among all immune clusters; IT index: relative abundance of immune and stromal clusters normalized to tumor clusters.

## Data Availability

Data associated with this study are provided in the Supplementary Information (Table S4). IMC images are not publicly available because of ethical and legal restrictions. De-identified data may be made available to qualified researchers upon reasonable request and with approval from the relevant ethics/data access authorities. Requests can be directed to REK Vest (rek-vest@uib.no), Norway (Rogaland and Vestland). A material transfer agreement (MTA) between Stavanger University Hospital and Erasmus MC is in place for the transfer of study materials.

## Supplementary Materials

The following supporting information can be downloaded at: https://www.mdpi.com/article/10.3390/cancers18060938/s1, Figure S1: H&E-stained sections used for histopathological review of IMC-based annotations; Figure S2: UMAP embedding of 82 high-risk NMIBC samples showing 18 phenotypic clusters annotated based on dominant marker expression; Figure S3: Cellular neighborhood composition based on aggregated marker expression; Figure S4: Heatmap showing summed significant interactions between cell types across 82 samples; Figure S5: Confusion matrix for the independent 10 patient test cohort (decision threshold = 0.5); Figure S6: Learning curves of the GA MIL model across three cross validation folds; Table S1 Recurrent cell–cell interaction pairs (FDR < 0.05) across the cohort (prevalence ≥ 60%); Table S2: Patient level predictions in the hold out set (true label and raw prediction score); Table S3: Patch-level classification performance change after individual channel removal; Table S4: Single-Cell IMC analysis dataset.

## Abbreviations

The following abbreviations are used in this manuscript:

BCG	Bacillus Calmette–Guérin
BRS	BCG response subtype
CAFs	Cancer-associated fibroblasts
CI	Confidence interval
CIS	Carcinoma in situ
CNN	Convolutional neural network
CNS	Cellular neighborhoods
EAU	European Association of Urology
FFPE	Formalin-fixed paraffin-embedded
FDR	False discovery rate
H&E	Hematoxylin and eosin
HL	Hodges-Lehmann
HR	Hazard ratio
ICTC	Immune cell cluster located within the tumor compartment
IHC	Immunohistochemistry
II index	Immune composition within the immune compartment
IMC	Imaging mass cytometry
IMC-GA-MIL	IMC-specific gated attention multiple instance learning
IT index	Immune/stromal abundance relative to tumor cells
kNN	k-nearest neighbors
LVI	Lymphovascular invasion
MIBC	Muscle-invasive bladder cancer
NK cells	Natural killer cells
NMIBC	Non-muscle-invasive bladder cancer
PFS	Progression-free survival
RFS	Recurrence-free survival
ResNet	Residual neural network
SCs	Spatial contexts
TMA	Tissue microarray
TME	Tumor microenvironment
TT index	Tumor phenotypic composition
Treg	Regulatory T cell
