# Supplementary material for: Spatial Immune Profiling and AI-Based Classifiers Identify Predictors of BCG Therapy Outcomes in High-Risk Non-Muscle-Invasive Bladder Cancer
Source: Cancers (Basel). 2026 Mar 13;18(6):938. doi: 10.3390/cancers18060938 (PMC13024979; doi:10.3390/cancers18060938)
Supplement: Supplementary file 1 [file cancers-18-00938-s001.zip › Supplementary information.pdf]

Supplementary information

Spatial immune profiling and AI-based classifiers identify predictors of BCG therapy outcome in high-risk non-muscle invasive bladder cancer

Melinda Lillesand[1,2,*], Marie Austdal[3], Jakub Mroz[4], Ivar Skaland[1], Einar Gudlaugsson[1], Florus C. de Jong[5], Tahlita C.M. Zuiverloon[5], Kjersti Engan[4], and Emiel AM Janssen[1,2]

1 Department of Pathology, Stavanger University Hospital, Stavanger 4011, Norway
2 Department of Chemistry, Bioscience and Environmental Engineering, University of Stavanger, Stavanger 4021, Norway
3 Section for Biostatistics, Department of Research, Stavanger University Hospital, Stavanger 4011, Norway
4 Department of Electrical Engineering and Computer Science, University of Stavanger, 4021 Stavanger, Norway
5 Department of Urology, Erasmus University Medical Center, Erasmus MC Cancer Institute, Rotterdam, the Netherlands
*Correspondence: melinda.lillesand@sus.no

Figure S1……………………………………………………………………………2
Figure S2……………………………………………………………………………3
Figure S3……………………………………………………………………………3
Figure S4……………………………………………………………………………4
Figure S5……………………………………………………………………………4
Figure S6……………………………………………………………………………5
Table S1……………………………………………………………………………5-6
Table S2……………………………………………………………………………6
Table S3……………………………………………………………………………6-7

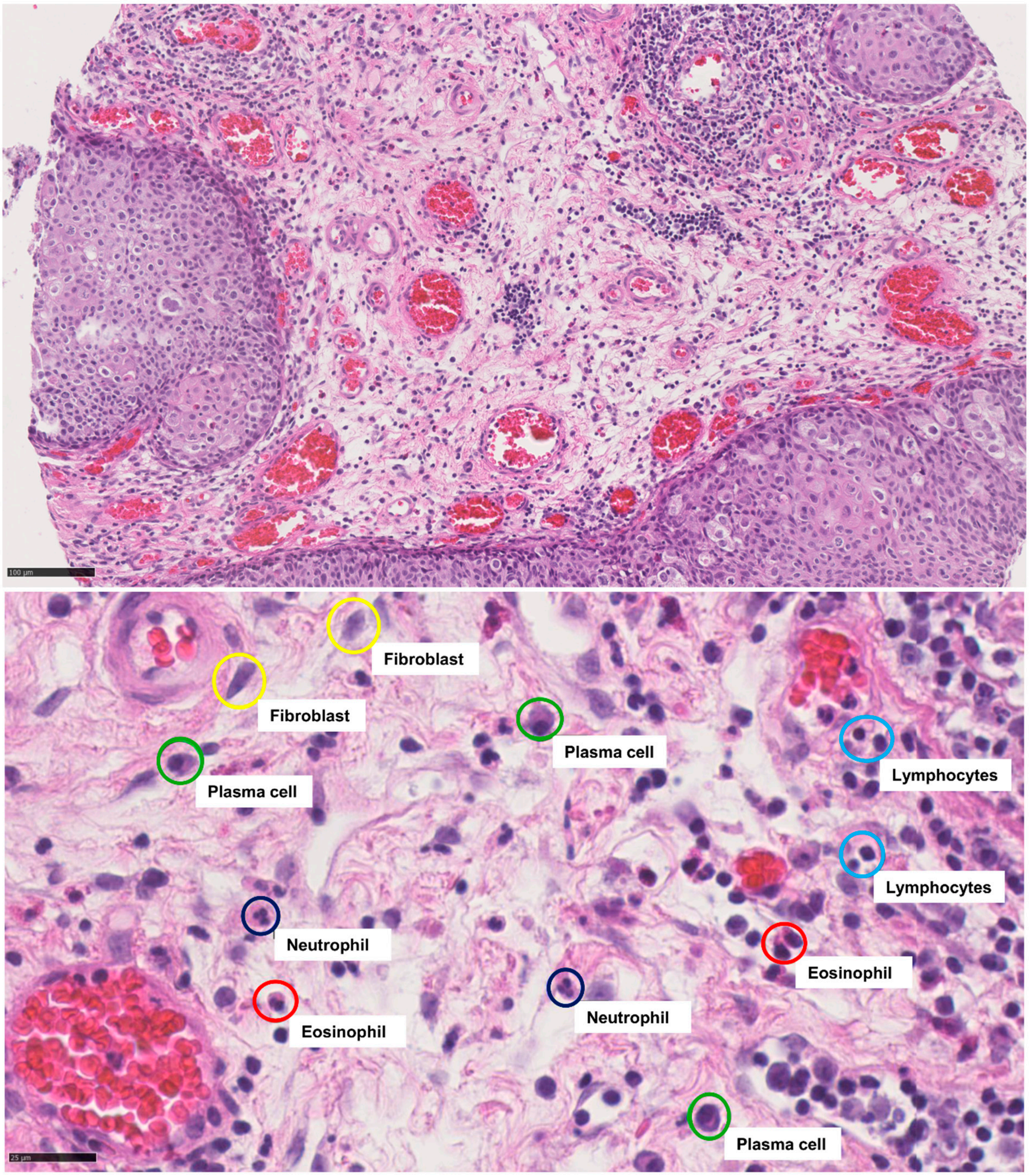

Figure S1. H&E-stained sections used for histopathological review of IMC-based annotations. The illustrated example is from patient P41. **Upper panel:** 200× magnification (scale bar: 100 µm). **Lower panel:** 800× magnification (scale bar: 25 µm).

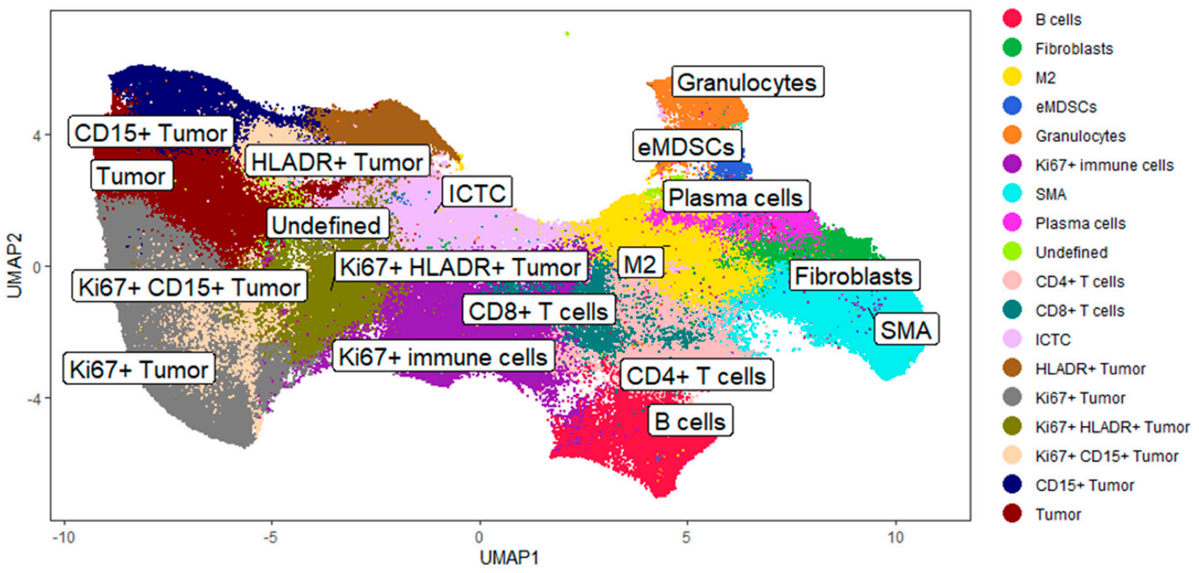

Figure S2. UMAP embedding of 82 high-risk NMIBC samples showing 18 phenotypic clusters annotated based on dominant marker expression.
Abbreviations: ICTC, immune cluster located within the tumor compartment.

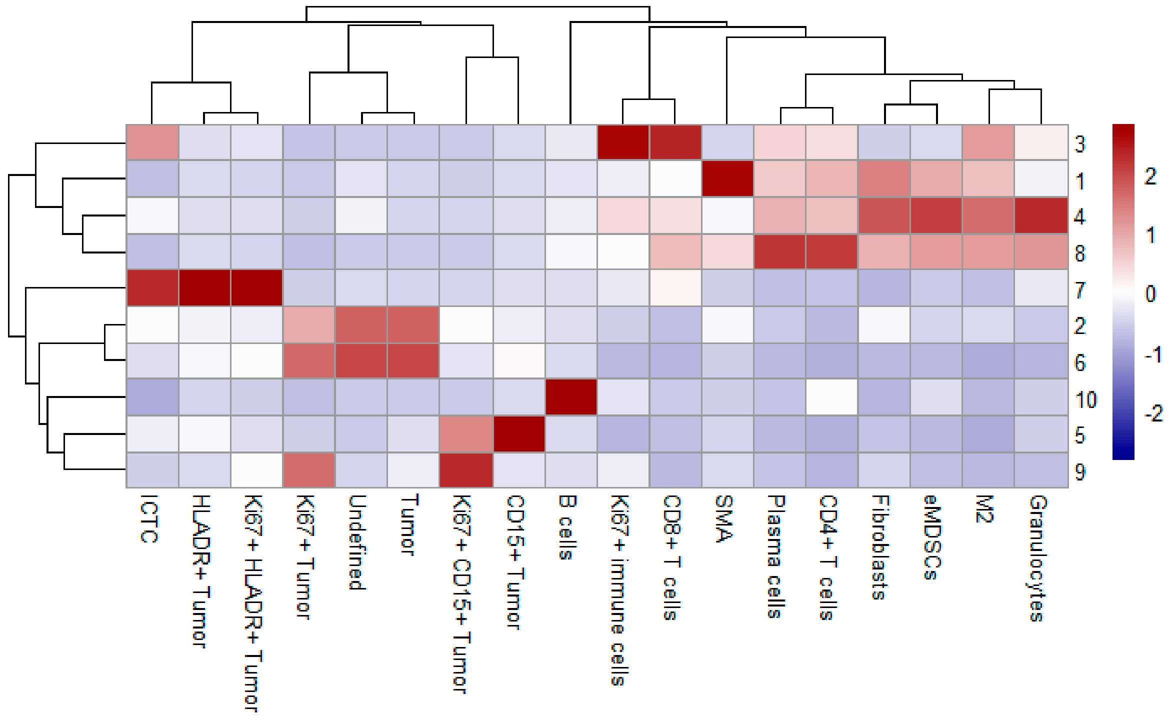

Figure S3. Cellular neighborhood composition based on aggregated marker expression. Heatmap showing the scaled mean marker expression across 10 cellular neighborhoods (rows) and 18 phenotypic clusters (columns). Red indicates higher, and blue lower, relative expression within each neighborhood (z-scored by column). Dendrograms represent hierarchical clustering of cellular neighborhoods and clusters based on similarity in expression profiles.
Abbreviations: ICTC, immune cluster located within the tumor compartment.

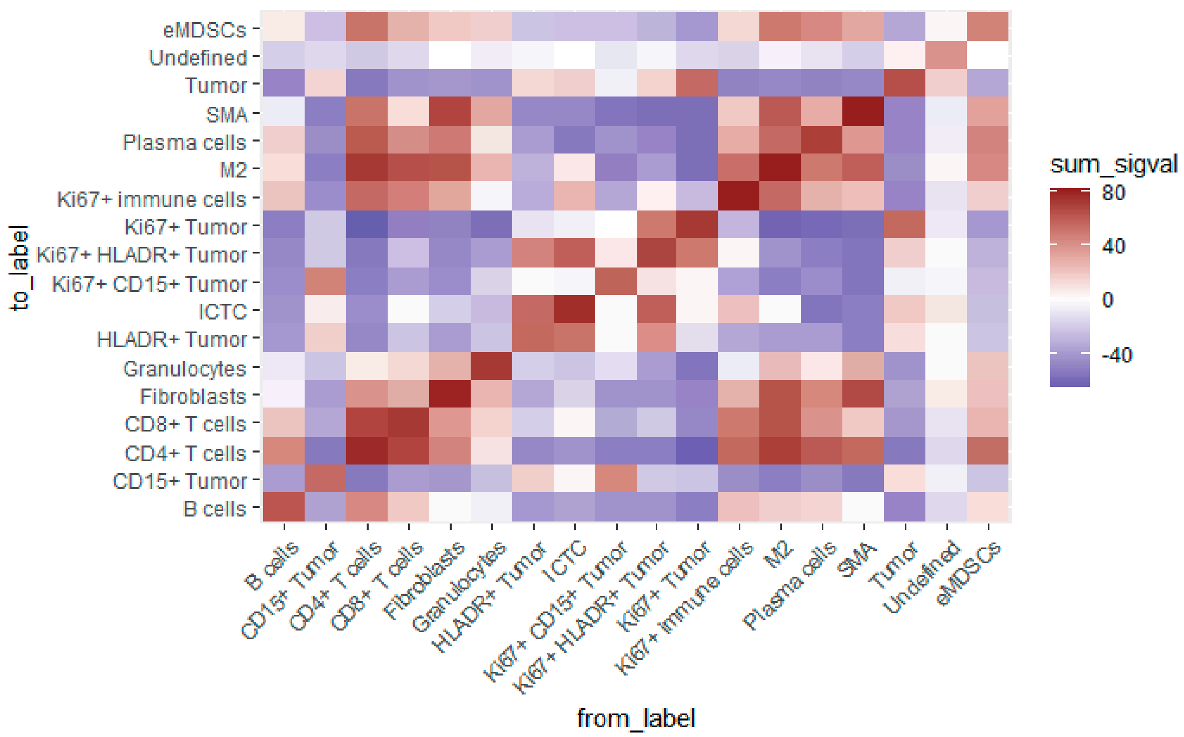

Figure S4. Heatmap showing summed significant interactions between cell types across 82 samples. Each square represents the direction and frequency of significant interactions (sum_sigval) between two cell populations. Red indicates increased interactions, blue indicates reduced interactions, and white indicates no consistent pattern across samples. Color intensity reflects the number of samples with a significant association, ranging from –82 (all negative) to +82 (all positive). Abbreviations: ICTC, immune cluster located within the tumor compartment.

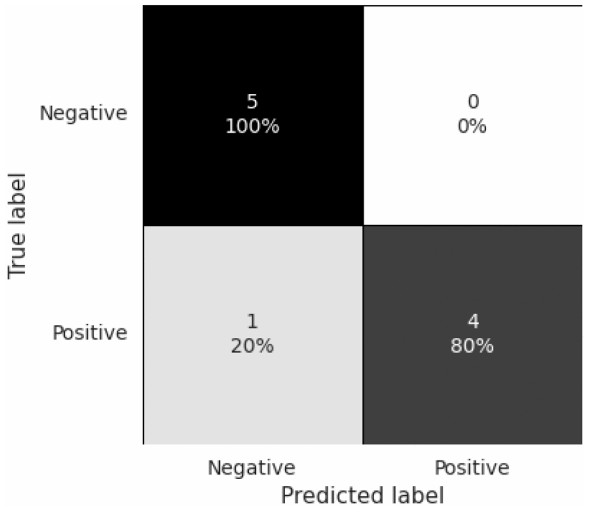

Figure S5. Confusion matrix for the independent 10-patient test cohort (decision threshold = 0.5). Cells show counts with within-row percentages.

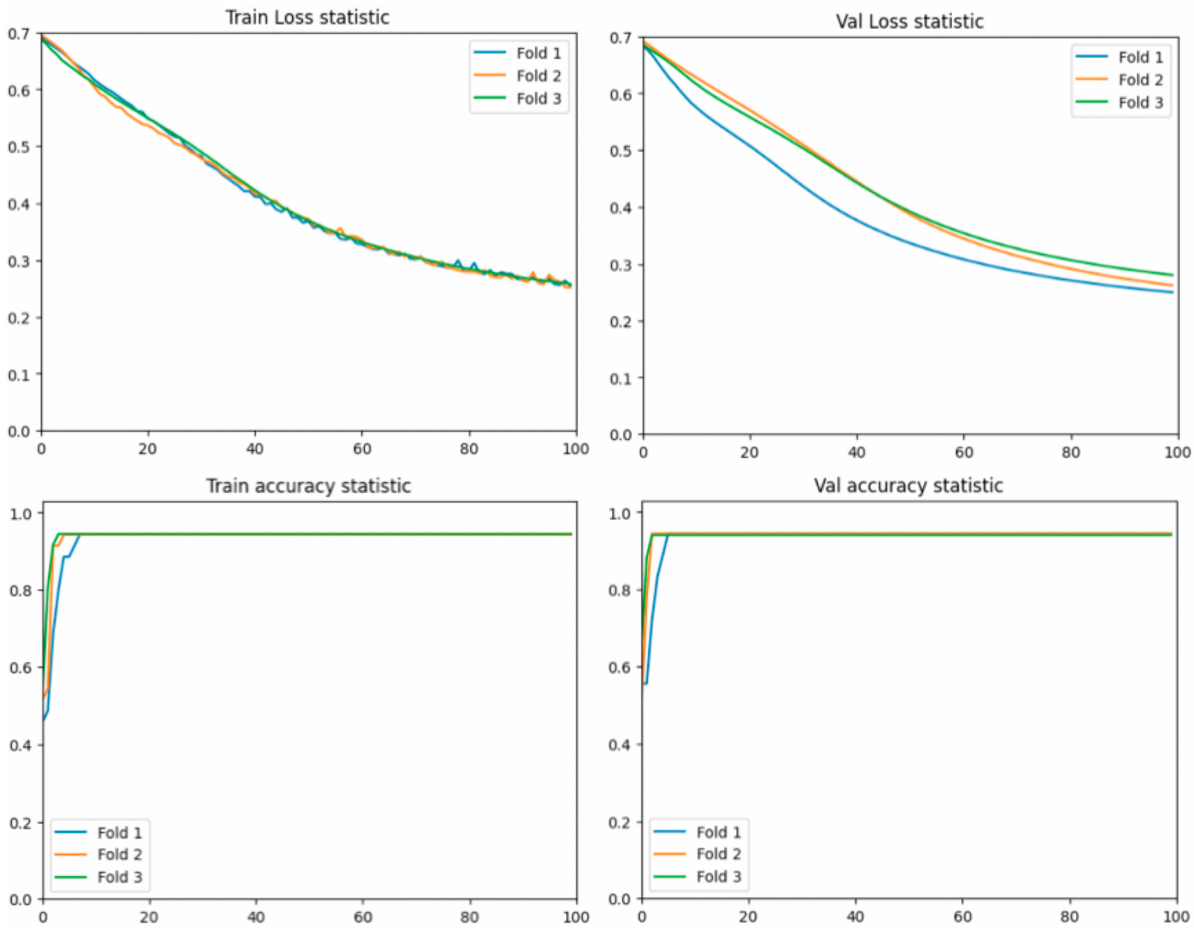

Figure S6. Learning curves of the GA-MIL model across three cross-validation folds. Top row: Training loss (left) and validation loss (right) plotted over 100 epochs for each fold. All folds show stable convergence and closely aligned training/validation trajectories, indicating limited overfitting. Bottom row: Training accuracy (left) and validation accuracy (right). Model accuracy saturates rapidly and remains stable throughout training, demonstrating consistent performance across folds and supporting that the model does not memorize training data.

Table S1. Recurrent cell–cell interaction pairs (FDR < 0.05) across the cohort (prevalence ≥ 60%).

Pair	Patients (n)	Prevalence	min FDR (q)	Mean ct	Median ct
CD4$^+$ T cells – M2 macrophages	75	0.91	0.002	2.67	2.53
Fibroblasts – α-SMA$^+$	74	0.90	0.002	3.49	2.99
CD4$^+$ T cells – CD8$^+$ T cells	71	0.87	0.002	1.36	1.26
CD8$^+$ T cells – M2 macrophages	69	0.84	0.002	2.52	2.38
M2 macrophages – α-SMA$^+$	68	0.83	0.002	3.22	2.80
CD4$^+$ T cells – α-SMA$^+$	67	0.82	0.002	3.17	2.90
Fibroblasts – M2 macrophages	67	0.82	0.002	2.48	2.32
CD4$^+$ T cells – Plasma cells	65	0.79	0.002	1.43	1.01
ICTC – Ki67$^+$ HLADR+ Tumor	65	0.79	0.002	1.11	0.73
Ki67$^+$ immune cells – M2 macrophages	64	0.78	0.002	2.25	2.08
CD4$^+$ T cells – Ki67+ immune cells	63	0.77	0.002	1.56	1.31
M2 macrophages – Plasma cells	63	0.77	0.002	1.11	0.75
Ki67$^+$ Tumor – Tumor	61	0.74	0.002	4.19	2.71

$Ki67^+$ $HLADR^+$ Tumor – $Ki67^+$ Tumor	60	0.73	0.002	3.47	2.94
$HLADR^+$ Tumor – ICTC	59	0.72	0.002	2.55	2.21
CD4+ T cells – eMDSCs	56	0.68	0.002	0.51	0.26
B cells – $CD4^+$ T cells	55	0.67	0.002	3.62	3.20
$CD8^+$ T cells – $Ki67^+$ immune cells	55	0.67	0.002	1.63	1.45
M2 macrophages – eMDSCs	53	0.65	0.002	0.36	0.27
$CD4^+$ T cells – Fibroblasts	52	0.63	0.002	1.27	1.01
Granulocytes – α-SMA^+	52	0.63	0.002	4.36	3.73
Fibroblasts – Plasma cells	51	0.62	0.002	1.40	1.08

Abbreviations: ct, contact/interaction score; FDR, false discovery rate (Benjamini–Hochberg adjusted p-value).

Table S2. Patient-level predictions in the hold-out set (true label and raw prediction score).

True label	Raw prediction
0	0.02
0	0.07
0	0.07
0	0.09
0	0.18
1	0.19
1	0.53
1	0.79
1	0.80
1	0.82

Table S3. Patch-level classification performance change after individual channel removal.

Removed channel	ΔPrecision	ΔRecall	ΔF1
**CD11b**	–0.072	–0.216	**–0.174**
**CD14**	–0.042	–0.456	**–0.330**
CD15	–0.029	–0.034	–0.031
CD163	–0.014	0.023	0.001
CD20	–0.011	0.008	–0.003
CD204	–0.003	–0.042	–0.025
CD278	–0.007	0.016	0.004
CD4	–0.024	–0.068	–0.056
CD56	0.024	–0.035	–0.007
**CD68**	–0.019	–0.181	**–0.110**
**CD8**	–0.041	–0.262	**–0.177**
**FOXP3**	–0.013	–0.299	**–0.192**
Granzyme B	–0.038	–0.105	–0.071
PanKeratin	–0.025	0.125	0.039
Tbet	–0.004	–0.163	–0.09
TGF-β	–0.128	0.027	–0.063

Vimentin	0.027	–0.109	–0.049
VISTA	–0.041	0.116	0.025

Performance changes are reported relative to patch-level performance with all 18 channels present.
